# Nucleic Acid‐Modified Nanoparticles for Cancer Therapeutic Applications

**DOI:** 10.1002/smll.202500843

**Published:** 2025-05-27

**Authors:** Yunlong Qin, Xinghua Chen, Itamar Willner

**Affiliations:** ^1^ The Institute of Chemistry The Hebrew University of Jerusalem Jerusalem 91904 Israel

**Keywords:** aptamers, chemodynamic, DNAzymes, gene therapy, G‐quadruplexes, photodynamic, photothermal

## Abstract

Nanomaterials including metal or metal oxide nanoparticles, carbonous nanomaterial (e.g., carbon dots) or metal–organic framework nanoparticles provide porous, catalytically active surfaces and functional interfaces for binding of ions or molecular agents. By the conjugation of nucleic acids to the nanoparticles, hybrid nanostructures revealing emerging multimodal catalytic/photocatalytic activities, high loading capacities, and effective targeted cell permeation efficacies are formed. The review article exemplifies the application of nucleic acid‐modified nanoparticles conjugates for therapeutic treatment of cancer cells. Stimuli‐responsive reconfiguration of nucleic acid strands and the specific recognition and catalytic function of oligonucleotides associated with porous, catalytic, and photocatalytic nanoparticles yield hybrid composites demonstrating cooperative synergistic properties for medical applications. The targeted chemodynamic, photodynamic, photothermal and chemotherapeutic treatment of cancer cells by the oligonucleotide/nanoparticle conjugates is addressed. In addition, the application of oligonucleotide/nanoparticle conjugates for gene therapy treatment of cancer cells is discussed.

## Graphical abstract

Nucleic acid‐modified nanoparticles act as functional hybrids for chemodynamic, photodynamic, photothermal, and gene therapy of cancer cells. These are exemplified with aptamer‐modified catalytic nanoparticles, photosensitizer‐oligonucleotide functionalized nanoparticles, drug‐loaded stimuli‐responsive nucleic acid‐caged nanoparticle carriers, and DNAzyme/siRNA/antisense oligonucleotide‐modified nanoparticle hybrids, generating cooperative multimodal treatment of cancer cells.

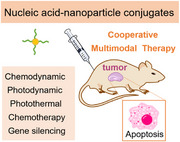

## 1 Introduction

The base sequence of nucleic acids encodes substantial structural and functional information into the biopolymers.^[^ ^1^, ^2^ ^]^ This includes sequence‐specific recognition of low‐molecular‐weight ligands and macrobiomolecular agents by aptamers^[^ ^3^, ^4^, ^5^, ^6^ ^]^ and sequence‐dictated catalytic functions by DNAzymes or ribozymes.^[^ ^7^, ^8^, ^9^, ^10^ ^]^ In addition, the base sequence of nucleic acids controls triggered structural reconfiguration of single strands into 3D structures, such as K^+^‐ion triggered formation of G‐quadruplexes^[^ ^11^, ^12^, ^13^ ^]^ or pH‐induced i‐motif structures.^[^ ^14^, ^15^ ^]^ The sequestered binding of low‐molecular‐weight agents or proteins to the reconfigured structures controls the functions of the guest ligands. For example, binding of porphyrin ligands (e.g., Fe(III)‐protoporphyrin) to G‐quadruplex, yields supramolecular catalytic complexes^[^ ^16^, ^17^, ^18^ ^]^ and the binding of the anti‐thrombin G‐quadruplex to thrombin inhibits the catalytic functions of thrombin.^[^ ^19^, ^20^ ^]^ Different applications of aptamers, DNAzymes and dynamically signal‐triggered reconfigurable oligonucleotides were reported. The sequence‐specific binding of aptamers were broadly applied to develop sensors,^[^ ^21^, ^22^ ^]^ to target therapeutic agents^[^ ^23^, ^24^, ^25^ ^]^ and to selectively inhibit proteins for therapeutic applications.^[^ ^26^, ^27^ ^]^ DNAzymes and ribozymes were used as catalytic labels for amplifying sensing events,^[^ ^28^, ^29^ ^]^ as catalysts for gene therapy,^[^ ^30^, ^31^ ^]^ and by conjugation of DNAzymes to aptamers, functional nucleic acids mimicking native enzymes (nucleoapzymes)^[^ ^32^, ^33^ ^]^ were developed. The dynamically reconfigurable properties of nucleic acids were broadly applied to develop DNA‐based machines^[^ ^34^, ^35^, ^36^, ^37^ ^]^ and switches,^[^ ^38^, ^39^, ^40^ ^]^ reconfigurable 2D and 3D DNA structures^[^ ^41^, ^42^, ^43^ ^]^ and to assemble DNA materials exhibiting switchable properties, such as switchable stiffness hydrogels and their uses for self‐healing,^[^ ^44^, ^45^ ^]^ shape‐memory^[^ ^46^, ^47^ ^]^ and actuating/robotic.^[^ ^48^, ^49^ ^]^

Inorganic nanoparticles (NPs), composed of metal, metal oxide, carbon‐based, semiconductor or metal‐organic framework (MOF) NPs, exhibit unique catalytic (nanozyme),^[^ ^50^, ^51^, ^52^, ^53^ ^]^ optical,^[^ ^54^, ^55^, ^56^ ^]^ plasmonic^[^ ^57^, ^58^ ^]^ and electronic functions.^[^ ^59^, ^60^ ^]^ Functionalization of nanoparticles with nucleic acids provides hybrid systems combining the unique features of the NPs and oligonucleotide constituents, and often emerging functions from the composite assemblies.^[^ ^61^ ^]^ Diverse applications of nucleic acid/nanoparticle hybrids in sensing,^[^ ^62^, ^63^ ^]^ optical,^[^ ^13^, ^64^ ^]^ catalytic^[^ ^65^ ^]^ and medical (imaging or therapeutic)^[^ ^66^, ^67^, ^68^ ^]^ fields were reported. For example, aggregation of nucleic acid‐modified plasmonic Au NPs and the emerging coupled interparticle plasmon exciton have been broadly applied for the development of sensing platforms^[^ ^69^ ^]^ and aptamer‐functionalized Au NPs^[^ ^70^ ^]^ or Cu^2+^‐C‐dots^[^ ^71^ ^]^ were used as enzyme‐mimicking assemblies (aptananozymes). Nucleic acid‐modified semiconductor quantum dots were used for developing diverse imaging and sensing platforms^[^ ^72^ ^]^ and the emerging optical functions in the hybrid nanostructures, e.g., chemiluminescence resonance energy transfer (CRET), were applied for multiplexed sensing of genes.^[^ ^73^ ^]^ In addition, nucleic acid‐modified plasmonic nanoparticles were used to engineer nanoscale optical devices, such as antenna^[^ ^74^, ^75^, ^76^ ^]^ or chiroplasmonic DNA machines,^[^ ^77^, ^78^ ^]^ and stimuli‐responsive nucleic acid/nanoparticle hybrids were applied to develop transient, dissipative, catalytic systems^[^ ^79^, ^80^ ^]^ and particularly stimuli‐triggered MOF^[^ ^81^ ^]^ or SiO_2_ NPs^[^ ^82^, ^83^ ^]^ for controlled drug release.

**Scheme 1:**
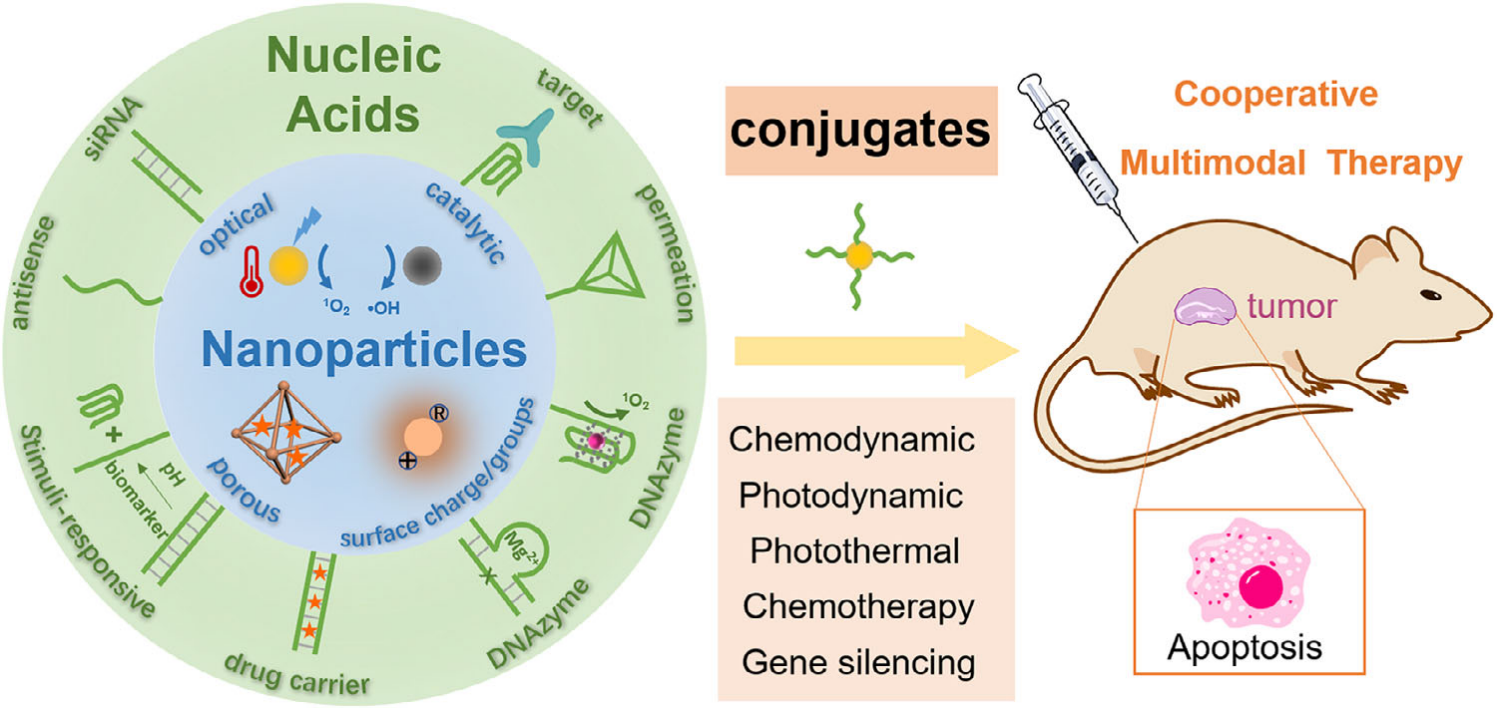
Schematic presentation of nucleic acid/nanoparticle conjugates for cooperative, multimodal cancer therapy.

The present review article discusses different approaches to apply nucleic acid/nanoparticle hybrids for cancer therapy, **Scheme** **1**. We exemplify the use of the hybrid systems for chemotherapy, chemodynamic, photodynamic, photothermal and gene therapy. In fact, substantial efforts were directed to the development of spherical nucleic acids (SNAs), where nucleic acids were self‐assembled in the form of spherical micelles, liposomes, protein/polymer anchored assemblies or integrated as oligonucleotide constituents on core inorganic particles. These structures were mainly used as hybrids for carrying and delivering the nucleic acids and their medical applications for diagnostic/sensing,^[^ ^63^, ^84^ ^]^ therapeutic^[^ ^85^ ^]^ and material assembly.^[^ ^86^, ^87^, ^88^ ^]^ While the topic of SNAs was extensively reviewed,^[^ ^89^, ^90^ ^]^ the distinct contributions of the present article should be noted. We emphasize the cooperative synergistic and multimodal functions of nucleic acid‐modified nanoparticles, while focusing on the applications of these hybrids for cancer therapy. We specifically emphasize the cooperative functions of the nucleic acid/nanoparticle conjugates for resolving fundamental issues related to cancer therapies. These include targeting of cancer cells and enhanced cell permeation of the treatment agents and therapeutic carriers into the cancer cells. Moreover, the catalytic, photocatalytic, photothermal, gene silencing properties and cooperative therapeutic effects of the nucleic acid/nanoparticle conjugates are addressed. In addition, the unique stimuli‐controlled drug release by the nucleic acid/nanoparticle carriers enhancing selectivity of the therapeutic platforms is addressed. Previous review articles^[^ ^91^, ^92^, ^93^ ^]^ addressed the application of DNA as a functional therapeutic material and discussed its integration within nanostructured frameworks as carriers. The present report emphasizes the cooperative and synergistic functions of the nucleic acid‐modified nanoparticle hybrids emerging from the combined physical and catalytic properties of the nanoparticle and the structural programmability of nucleic acids and their unique recognition or catalytic functions.

## 2 Aptamer‐Modified Nanoparticles for Cancer Therapy

Aptamers are sequence‐specific nucleic acids recognizing low‐molecular‐weight ligands or macromolecules, elicited by the systematic evolution of ligands by exponential enrichment (SELEX) procedure.^[^ ^94^, ^95^ ^]^ Conjugation of the aptamer to catalytically active particles, photocatalytically active or photothermal active particles provides versatile means to target the particles to specific cancer cells receptor, enhance their cell permeation and localize the nanoparticle.

### 2.1 Chemodynamic Treatment of Cancer Cells by Aptamer‐Modified Catalytic Nanoparticles

**Figure 1:**
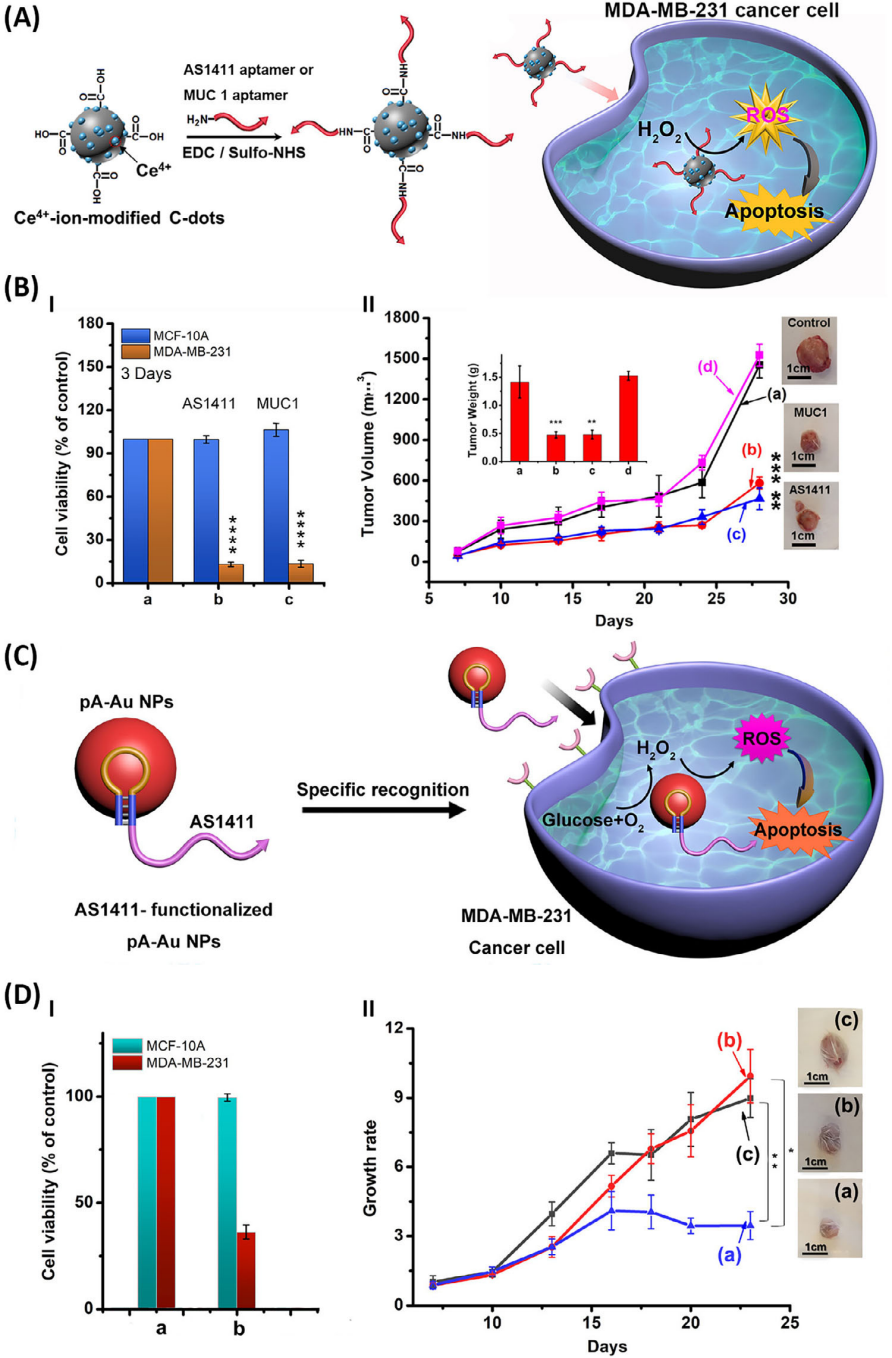
A) Schematic synthesis of Ce^4+^‐ion‐modified C‐dots functionalized with the AS1411 anti‐nucleolin aptamer or the anti‐MUC‐1 aptamer and their use for the catalyzed generation of ROS cytotoxic agents against cancer cells. B) Panel I‐Cytotoxicity of the aptamer‐functionalized Ce^4+^‐modified C‐dots (5 µg mL^−1^, 3 days) toward MDA‐MB‐231 breast cancer cells, in comparison to non‐cancerous MCF‐10A epithelial breast cells. Panel II‐Inhibition of MDA‐MB‐231 tumor growth in the presence of: (a) control with Ce^4+^‐modified C‐dots only, (b) AS1411 aptamer‐functionalized Ce^4+^‐modified C‐dots, (c) MUC‐1 aptamer‐functionalized Ce^4+^‐modified C‐dots, (d) control with PBS buffer. C) Schematic use of AS1411 aptamer conjugated polyadenine‐functionalized Au (pA‐Au) NPs as catalysts for the generation of cytotoxic ROS agents in cancer cells. D) Panel I‐Cytotoxicity of the AS1411 aptamer‐modified pA‐Au NPs toward MDA‐MB‐231 breast cancer cells in comparison to normal epithelial MCF‐10A cells: (a) control system of non‐treated cells, (b) cells treated with the AS1411 pA‐modified Au NPs, 1.5 nM for 2 days. Panel II‐Growth profiles of the MDA‐MB‐231 tumor treated with: (a) AS1411/pA‐Au NPs, (b) pA‐Au NPs, (c) control of randomized AS1411/pA‐Au NPs. A,B) Reproduced under terms of the CC‐BY 4.0 license.^[^ ^99^ ^]^ Copyright 2022, ACS. C,D) Reproduced under terms of the CC‐BY 4.0 license.^[^ ^70^ ^]^ Copyright 2022, ACS.

Diverse nanozymes reveal peroxidase mimicking functions generating ·OH or $O_{2}^{-\cdot }$ radicals as toxic reactive oxygen species (ROS) leading to apoptosis of cells.^[^ ^96^, ^97^, ^98^ ^]^ Nonetheless, the non‐specific ROS generation by the nanozymes represents a key limitation in their chemodynamic application toward targeting cells. The anchoring of sequence‐specific aptamers targeting receptors associated with cancer cells to nanozyme agents provide versatile means to localize the aptamer/nanozyme hybrids at target cancer cells, thereby enhancing the selectivity of chemodynamic treatment of the cancer cells. This is exemplified in **Figure** **1**A with the synthesis of Ce^4+^‐modified carbon dots (Ce^4+^‐C‐dots) functionalized with the anti‐nucleolin receptor AS1411 aptamer or the anti‐MUC‐1 receptor aptamer.^[^ ^99^ ^]^ The AS1411 aptamer ‐modified Ce^4+^‐C‐dots or the MUC‐1 aptamer modified Ce^4+^‐C‐dots targets the nucleolin or MUC‐1 receptor associated with MDA‐MB‐231 breast cancer cells, thereby confining the ROS generating efficacies for the localized chemodynamic therapeutic treatment of MDA‐MB‐231 breast cancer cells or MDA‐MB‐231 tumors elicited in xenograft mice, Figure 1B, Panel I and Panel II. In addition, AS1411 aptamer tethered polyadenine (pA) stabilized Au NPs were applied as aptamer‐functionalized nanozymes for selective in vitro and in vivo chemodynamic treatment of MDA‐MB‐231 breast cancer cells and tumors.^[^ ^70^ ^]^ The pA‐stabilized Au NPs reveal cascaded nanozyme activities where Au NPs catalyze the aerobic oxidation of glucose to gluconic acid and H_2_O_2_, and the subsequent catalyzed generation of ·OH as ROS cytotoxic agent, Figure 1C. The targeted aptamer guided generation of the cytotoxic ·OH in the cancer cells led to selective in vitro cytotoxicity, Figure 1D, Panel I, and effective tumor inhibition growth in MDA‐MB‐231 tumor‐bearing mice, Figure 1D, Panel II.

### 2.2 Photodynamic Treatment of Cancer Cells by Aptamer/Nanoparticle Hybrids

Photosensitizers are useful agents for photodynamic therapy of cancer cells through the generation of reactive toxic products degrading cells by interfering in cellular pathways to destroy protein, sugar, or nucleic acid.^[^ ^102^ ^]^ The photosensitizer‐induced transformation leading to the reactive toxic intermediates include the photosensitized transition of triplet oxygen (^3^O_2_) to toxic singlet oxygen (^1^O_2_) or the photosensitized electron transfer to triplet oxygen yielding superoxide radical ($O_{2}^{-\cdot }$), being further enzymatically transformed into H_2_O_2_ that provides a substrate for secondary generation of hydroxyl radicals (·OH).^[^ ^103^, ^104^, ^105^ ^]^ This set of ROS provides not only a chemical arsenal of toxic intermediates destroying biomaterial, but also mediating agents for generating other toxic agents,^[^ ^106^, ^107^ ^]^ such as peroxynitrite (ONOO^–^) from endogenous nitric oxide, or ${\mathrm{CO}}_{3}^{-\cdot }$ from endogenous bicarbonate. Beyond the capacities of photosensitizers to generate reactive toxic agents, the photoactivity of plasmonic nanoparticles results in photo‐driven heating,^[^ ^108^, ^109^ ^]^ allowing cooperative photodynamic and photothermal degradation of cells. Nevertheless, the photosensitized or plasmonic‐induced degradation of biological tissues is non‐selective and accompanied by cytotoxicity toward healthy organs. The targeting of the photosensitizing agents to specific cancer cells by means of aptamer units, provides a versatile mechanism for spatiotemporal light‐activated photodynamic therapy, and this will be exemplified in this section.

**Figure 2:**
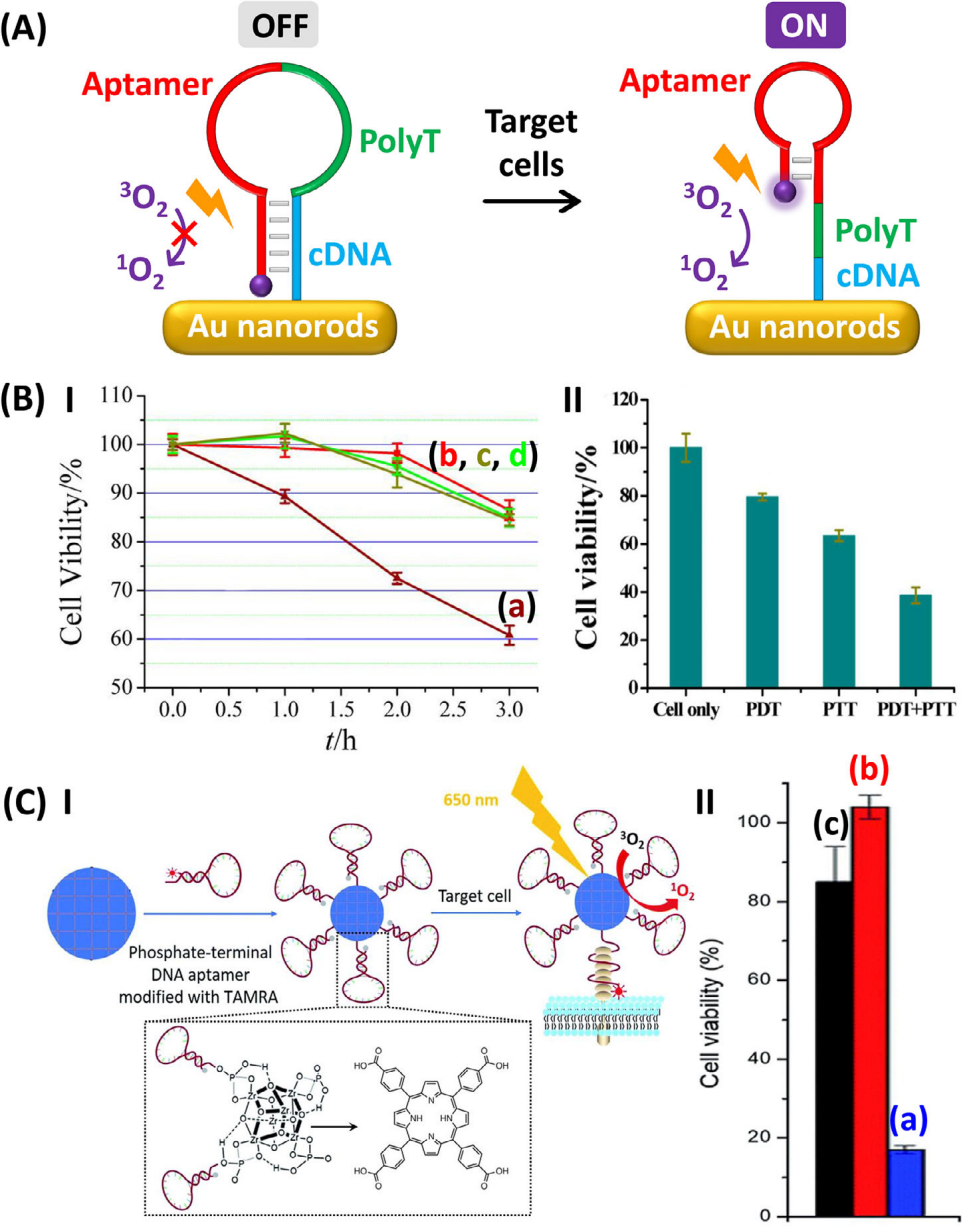
A) Schematic application of anti‐protein tyrosine kinase‐7 (PTK‐7) aptamer engineered, chlorin e6‐modified hairpin linked Au nanorods for the cooperative photodynamic and photothermal treatment of CCRF‐CEM leukemia T‐cells. B) Panel I‐Time‐dependent cell viability upon: (a)‐(b) treatment of PTK‐7‐associated CCRF‐CEM leukemia T‐cells with (a) and without (b) functionalized Au nanorods under light (λ_ex_ = 660 nm), (c)‐(d) treatment of non‐PTK‐7‐associated Ramos leukemia B‐cells with (c) and without (d) functionalized Au nanorods under light (λ_ex_ = 660 nm). Panel II‐Cell viability of PTK‐7‐associated CCRF‐CEM leukemia T‐cells upon photodynamic (PDT, λ_ex_ = 660 nm), photothermal (PTT, λ_ex_ = 812 nm) and cooperative PDT and PTT treatment. C) Panel I‐Assembly of porphyrin‐bridged, anti‐PTK‐7 aptamer engineered MOF particles for the photodynamic treatment of HeLa cancer cells. Panel II‐HeLa cell viability of upon: (a) treatment of the cells with the anti‐PTK‐7 aptamer‐modified MOF particles under light (λ_ex_ = 650 nm), (b) treatment with the anti‐PTK‐7 aptamer‐modified MOF particles in the absence of light, (c) treatment with random sequence‐modified MOF particles under light (λ_ex_ = 650 nm). (A)‐(B) Reproduced with permission.^[^ ^100^ ^]^ Copyright 2012, ACS. (C) Reproduced under terms of a Creative Commons Attribution 3.0 Unported Licence.^[^ ^101^ ^]^ Copyright 2018, RSC.

**Figure** **2**A depicts the application of Au nanorods functionalized with a photosensitizer chlorin e6 (Ce6)‐modified hairpin that includes in its loop/stem domain a caged aptamer sequence recognizing the membrane protein tyrosine kinase‐7 (PTK‐7) associated with lymphoblastic leukemia T‐cells for the targeted treatment of cancer cells.^[^ ^100^ ^]^ The spatial proximity of the photosensitizer leads to quenching of the photosensitizer and to a photodynamically inactive unit. The targeting of the photodynamic carrier to the cancer cells via formation of the aptamer/PTK‐7 complex, opens the hairpin into a configuration spatially separating the photosensitizer from the Au nanorods. This results in the activation of the photodynamic function of the photosensitizer, leading to the generation of ^1^O_2_ cytotoxic agents in proximity of the cancer cells. Moreover, the plasmonic feature of the Au nanorods (λ_ex_ = 812 nm) enables the cooperative photothermal degradation of the cancer cells. Figure 2B demonstrates the targeted photodynamic treatment of the cancer cells and the cooperative photothermal effect on the cell viability. Irradiation of the target leukemia T‐cells with white light in the presence of the hairpin‐modified Au nanorods leads to a 40% decrease in the cell viability upon irradiation for a time interval of 3 h, whereas control experiments involving the irradiation of non‐PTK‐7 containing Ramos cells or leukemia T‐cells in the absence of the photosensitizer functionalized carrier reveal substantially lower degrease (15%) of cell death under similar conditions, Panel I. The separate and cooperative photodynamic/photothermal effects of the carrier on the leukemia T‐cells viability are displayed in Panel II. While the photodynamic treatment for 2 h leads to 20% cell death and the photothermal effect (λ_ex_ = 812 nm) leads to ca. 40% cell death, the irradiation of the cells with the two light sources leads to ca. 60% cell death. In a related study,^[^ ^101^ ^]^ MOF particles composed of the photosensitizer meso‐tetra(4‐carboxyphenyl)porphine bridged Zr^4+^‐framework functionalized with caged PTK‐7 aptamer hairpin structure were employed as photosensitizer units for photodynamic treatment of PTK‐7 containing HeLa cells. Upon irradiation of the HeLa cells with a light source (650 nm, 200 mW · cm^−2^) for 5 min, ca. 80% cell death was observed, while particles modified with a non‐aptamer functionalized hairpin revealed only 15% of cell death under similar conditions, demonstrating the spatial and selective photodynamic treatment, induced by the aptamer‐modified framework particles, Figure 2C.

### 2.3 Photothermal Treatment of Cancer Cells by Aptamer‐Modified Light‐Heating Nanoparticles

Light‐stimulated photothermal heating of cancer cells by aptamer‐modified nanoparticle agents was further applied to selectively control cell viability. Three general mechanisms to induce photothermal effects were reported.^[^ ^110^ ^]^ One includes plasmonic localized heating where the photoexcitation of surface plasmon electrons coupled with electron‐phonon scattering leads to non‐radiative decay of the photoexcited plasmonic electrons.^[^ ^111^, ^112^ ^]^ The second mechanism involves non‐radiative relaxation of photoexcited electron‐hole pairs in semiconductor nanoparticle agents,^[^ ^113^ ^]^ and the third mechanism includes thermal dissipation of photoexcited vibration energy states in carbonaceous^[^ ^114^ ^]^ or polymer^[^ ^115^ ^]^ materials.

**Figure 3:**
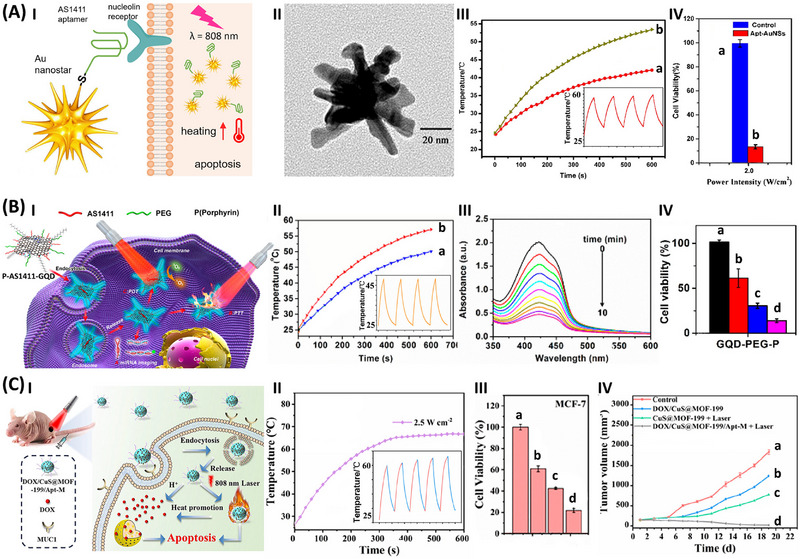
A) Panel I‐Schematic application of AS1411 aptamer‐modified plasmonic Au nanostar particles for the photothermal treatment of cancer cells. Panel II‐TEM image of the Au nanostar particles. Panel III‐Temporal temperature changes upon photoexcitation (λ_ex_ = 808 nm) using different concentrations of the particles: (a) 30 µg mL^−1^, (b) 150 µg mL^−1^. Inset switchable temperature changes of the solution upon the ON/OFF photoexcitation of the particles. Panel IV‐Photothermal effect on the HeLa cell viability upon treatment of the cells with the Au nanostar particles, 150 µg mL^−1^, without exposure to light (blue, a) and upon photothermal heating (808 nm, 10 min) (red, b). B) Panel I‐Schematic application of AS1411 aptamer‐functionalized graphene quantum dots modified with a porphyrin for the cooperative photothermal and photodynamic treatment of cancer cells. Panel II‐Photoinduced temperature changes of an aqueous solution in the presence of the modified graphene quantum dots: (a) 50 µg mL^−1^, (b) 200 µg mL^−1^. Inset‐Switchable temperature change upon ON/OFF illumination (λ_ex_ = 980 nm). Panel III‐Temporal photodynamic generation of ROS agents upon photoexcitation of the modified graphene quantum dots, λ_ex_ = 635 nm. Panel IV‐A549 cancer cell viability: (a) control system without irradiation, (b) irradiation at 635 nm, activating the photodynamic treatment, (c) irradiation at 980 nm, activating the photothermal treatment, (d) cooperative treatment of the cancer cells by simultaneous irradiation at 635 nm and 980 nm. C) Panel I‐Schematic cooperative photothermal and chemotherapeutic treatment of MCF‐7 cells/tumors using doxorubicin (DOX)‐loaded and CuS‐loaded MOF‐199 NMOFs. Panel II‐Temporal temperature changes of an aqueous solution upon illumination of the particle, λ_ex_ = 808 nm, and switchable temperature changes upon ON/OFF irradiation of the particles. Panel III‐MCF‐7 cell viability and Panel IV‐MCF‐7 tumor growth profile upon: (a) control system without particle treatment, (b) the DOX/CuS‐loaded particles under dark conditions, (c) the MOF particles loaded with only CuS under light photothermal treatment, (d)cooperative photothermal and chemotherapeutic treatment by the DOX/CuS‐loaded particles. (A) Reproduced with permission.^[^ ^116^ ^]^ Copyright 2018, Springer Nature. B) Reproduced with permission.^[^ ^118^ ^]^ Copyright 2017, ACS. C) Reproduced with permission.^[^ ^119^ ^]^ Copyright 2024, ACS.

**Figure** **3**A, Panel I exemplifies the application of Au nanostars modified with thiolated AS1411 aptamer for the photothermal treatment of HeLa cancer cells.^[^ ^116^ ^]^ The particles, Panel II, exhibit a surface plasmon resonance phenomenon in the NIR region, revealing thermoplasmonic effect controlled by the concentration of the nanoparticles and power of excitation energy, Panel III. The photothermal effect is switchable, Panel III inset. The photothermal effect of the AS1411 aptamer‐modified Au nanostars on the HeLa cell viability, as compared to control system irradiated in the absence of the particles, is displayed in Panel IV. While the control sample, excluding the plasmonic particles, was not affected by the light, the cancer cells treated with the aptamer‐modified thermoplasmonic particles demonstrated ca. 90% cell death after irradiation for 10 min with a light power source of 2.0 W cm^−2^. The effective photothermal treatment of the cancer cells was attributed to the spatial targeting and localization of the photothermal particles at the cancer cells by means of the aptamer constituent. Related studies demonstrated the application of AS1411 aptamer‐modified SiO_2_‐coated Au nanocages for the imaging and photothermal treatment of MCF‐7 breast cancer cells.^[^ ^117^ ^]^

The combined synergistic photothermal and photodynamic treatment of cancer cells is displayed in Figure 3B, Panel I.^[^ ^118^ ^]^ Graphene quantum dots (GQDs) are passivated with polyethylene and modified with AS1411 aptamer and hydrophobic porphyrin. While the GQDs provide the photothermal generation agents (λ_ex_ = 980 nm), Panel II, the porphyrin constituent provides photosensitizer units (λ_ex_ = 635 nm) for the photochemical generation of ROS (Panel III), and the AS1411 aptamer acts as cancer cell target agents. The effective synergistic dual‐wavelength photothermal/photodynamic treatment of cancer cells by the composite hybrid framework is displayed in Panel IV. While irradiation of A549 cancer cells with the hybrid at 635 nm for 10 min led to 50% cell death originating from the photosensitized ^1^O_2_ photodynamic mechanism, illumination of the cells using the photothermal effect (980 nm) led to 75% cell death, and illumination of the cells with dual light sources (635 nm + 980 nm) resulted in synergistic cell death corresponding to ca. 90%.

Moreover, the combined photothermal/chemotherapeutic treatment of MCF‐7 breast cancer cells is exemplified in Figure 3C, Panel I, using the MUC‐1 aptamer‐functionalized MOF nanoparticles (NMOFs)^[^ ^119^ ^]^ loaded with photoactive CuS semiconductor quantum dots and chemotherapeutic drug, doxorubicin (DOX). Cu^2+^‐ions and benzene tricarboxylic acid are employed to construct the MOF‐199 NMOFs by a solvothermal method. The functionalization of the particles with thioacetamide followed by in situ vulcanization generates CuS semiconductor dopants. Loading of the NMOFs with DOX and covalent functionalization of the particles with amine‐modified MUC‐1 aptamer yield the hybrid composites, where the CuS dots acts as the photothermal agent, Panel II (λ_ex_ = 808 nm), the DOX acts as therapeutic drug, and the aptamer tethers act as cancer cells targeting agents. The cooperative photothermal/chemotherapeutic in vitro treatment of MCF‐7 cells, Panel III, or in vivo treatment of MCF‐7 tumors in xenograft mice, Panel IV, were explored. While the non‐illuminated DOX‐loaded framework nanoparticles demonstrate 40% cell death after 4 h, the illuminated (λ_ex_ = 808 nm, 10 min) DOX‐free framework as photothermal heater reveals ca. 50% MCF‐7 cell death, and the photothermal irradiated cells subjected to the concomitant DOX release reveal ca. 80% cell death. Similarly, the in vivo experiment, Panel IV, reveals that non‐treated tumors show a rapid growth corresponding to 2000 mm^3^ within 19 days, whereas the non‐illuminated system subjected to the release of the drug only, shows tumor inhibition to 1200 mm^3^, the DOX‐free particles exhibit under photothermal treatment tumor inhibition to 700 mm^3^, and the tumor growth is fully inhibited upon the cooperative photothermal and chemotherapeutic treatment. Related experiments employing drug‐loaded, AS1411 aptamer‐modified GO, or aptamer‐functionalized Prussian Blue nanoparticles were reported as functional nanoparticles for the cooperative photothermal/chemotherapeutic(chemodynamic) targeted treatment of cancer cells.^[^ ^120^, ^121^ ^]^

Whilst the recognition functions of aptamers with cell‐membrane receptors provide a useful tool to target the nanoparticles to specific cells and assist cell permeability thereby controlling therapeutic selectivity, the limited binding affinities of aptamers, as compared to antibodies, must be acknowledged. Indeed, recent efforts are directed to improve the binding affinities by synthetic^[^ ^122^, ^123^, ^124^ ^]^ and computational^[^ ^125^ ^]^ means.

## 3 Photosensitizer/G‐Quadruplex or DNAzyme‐Modified Nanoparticles for Catalytic and Photocatalytic Cancer Therapy

The base sequence encoded in nucleic acids dictates binding interactions with metal ions, resulting in emerging catalytic and photocatalytic features in the supramolecular DNA/metal ion complexes. For example, the interaction of metal ions with sequence specific nucleic acids yields catalytic DNA structures (DNAzymes) revealing hydrolytic or ligation functions.^[^ ^126^ ^]^ In addition, sequence‐dictated oligonucleotides, for example, guanosine (G)‐rich nucleic acids, fold, in the presence of metal ions, such as K^+^, Sr^2+^, or Pb^2+^, into G‐quadruplex structures.^[^ ^127^, ^128^, ^129^ ^]^ The interaction of G‐quadruplex with π‐rich macrocycles, for example, porphyrins or metalloporphyrins, yields supramolecular structures revealing catalytic^[^ ^16^, ^17^, ^18^, ^130^ ^]^ or photocatalytic^[^ ^131^ ^]^ properties. For example, the binding of Fe(III)‐protoporphyrin IX (hemin) to G‐quadruplex is known to yield supramolecular peroxidase mimicking DNAzyme catalyzing the H_2_O_2_ oxidation of dopamine or NADH substrates,^[^ ^132^ ^]^ and other metalloporphyrins associated with G‐quadruplex catalyze reactions such as the Diels‐Alder process.^[^ ^133^ ^]^ Also, binding of photoactive metalloporphyrins/porphyrins photosensitizers to G‐quadruplex yields supramolecular complexes revealing photophysical and photocatalytic properties. For example, the binding of Zn(II)‐protoporphyrin IX (Zn(II)PPIX) to G‐quadruplex yields photosensitizer/DNA complex revealing enhanced fluorescence^[^ ^131^ ^]^ and superior photocatalytic^[^ ^134^ ^]^ properties. Coupling of the catalytic or photocatalytic nucleic acid structures to nanoparticle units does not only yield hybrid carriers for the catalyst/photocatalyst/DNAzyme constituents, but also enable chemical interactions between the nanoparticles and the DNAzyme units leading to synergistic catalytic/photocatalytic efficacies. In this section, the functions of photosensitizer/G‐quadruplex or DNAzyme‐modified particle hybrids for enhanced photodynamic and gene regulation cancer therapies will be exemplified.

### 3.1 Photosensitizer/G‐Quadruplex Modified Nanoparticles for Photocatalytic Cancer Therapy

**Figure 4:**
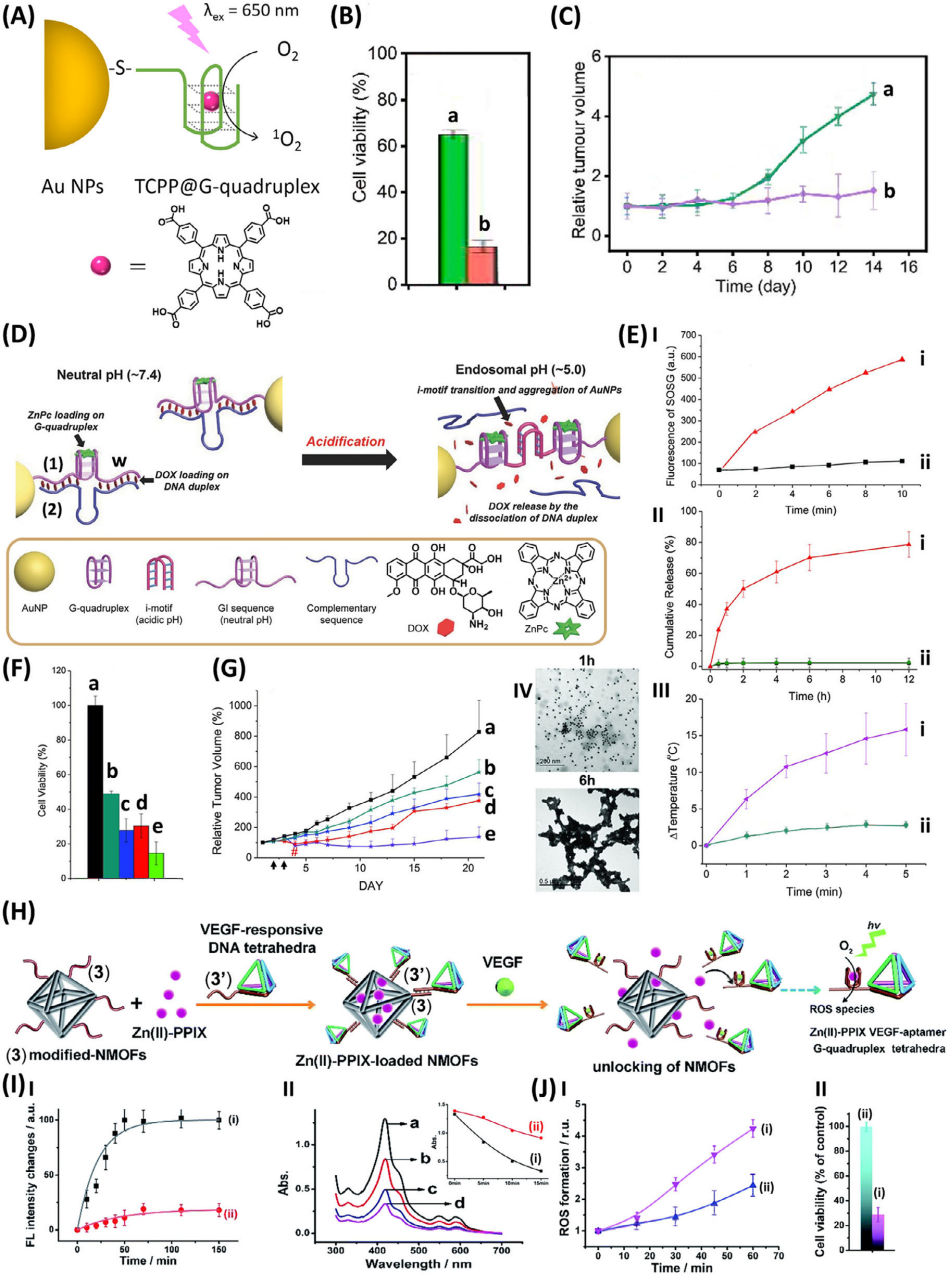
A) Assembly of a porphyrin‐loaded G‐quadruplex linked to Au NPs for the photodynamic therapeutic degradation of cancer cells by the photosensitized generation of ^1^O_2_. B) HeLa cell viability upon treatment with control systems: (a) the porphyrin‐loaded G‐quadruplex‐modified Au NPs in the dark, (b) the porphyrin‐loaded Au NPs under light/O_2_. C) In vivo inhibition of HeLa tumor growth in xenograft mice using control systems: (a) buffer, (b) porphyrin‐loaded G‐quadruplex‐modified Au NPs in the dark, (c) porphyrin‐loaded G‐quadruplex‐modified Au NPs under light (λ_ex_ = 650 nm). D) Schematic assembly of Au NPs functionalized with the duplex (1)/(2) for the cooperative treatment of cancer cells. Strand (1) includes the Zn(II) phthalocyanine, ZnPc modified G‐quadruplex and an extended tether “w” composed of C‐rich domain (being a subunit of the i‐motif structure at acidic pH values. Hybridization of strand (2) yields the hybrid (1)/(2) that binds the doxorubicin as intercalators into the DNA duplex. The resulting construct allows the multimodal treatment of cancer cells: (i) under light the ZnPc photodynamic treatment, (ii) at acidic pH‐values present in cancer cells, the separation of the duplex, releasing the chemotherapeutic DOX drug and the concomitant pH‐stimulated aggregation of Au NPs through i‐motif bridging units allowing the photothermal treatment of the cancer cells. E) Panel I‐ROS ^1^O_2_ generated by (i) non‐irradiated (1)/(2)‐modified Au NPs and (ii) irradiated (λ_ex_ = 660 nm) (1)/(2)‐modified Au NPs. Panel II‐The DOX release (i) at pH 7.4 and (ii) upon separation of the (1)/(2) duplex at pH 5.0. Panel III‐The photothermal properties of: (i) Non‐aggregated Au NPs at pH 7.4, (ii) Aggregated Au NPs at acidic pH (pH 5.0). Panel IV‐TME images corresponding to the pH‐stimulated aggregation of the Au NPs. F) MDA‐MB‐231 breast cancer cell viability upon treated with: (a) pure buffer solution, (b) the functionalized Au NPs (in the absence of irradiation, only DOX release), (c) irradiated at 650 nm (subjected to DOX release and photodynamic treatment), (d) irradiated at 808 nm (subjected to photothermal and DOX treatment), (e) irradiated at 650 nm and 808 nm (subjected to multimodal cooperative photodynamic/photothermal/DOX treatment. G) In vivo MDA‐MB‐231 tumor growth inhibition by the multi‐modal modified Au NPs, (a)‐(e) as in (F). H) Schematic synthesis of Zn(II)PPIX‐loaded UiO‐66 NMOFs gated by VEGF‐responsive aptamer‐modified DNA tetrahedra nanostructure for enhanced cell permeation, and VEGF‐driven unlocking of the NOMFs releasing Zn(II)PPIX for photodynamic generation of cytotoxic ^1^O_2_ species. I) Panel I‐Temporal release of the Zn(II)PPIX load: (i) in the presence of VEGF, 2 µM, (ii) in the absence of VEGF. Panel II‐Temporal absorbance changes of the DPBF probe monitoring the VEGF‐induced release of Zn(II)PPIX generating ^1^O_2_. Inset: (i) in the presence of VEGF, (ii) in the absence of VEGF. J) Panel I‐Integrated temporal ROS (^1^O_2_) generated in (i) MDA‐MB‐231 cancer cells and (ii) MCF‐10A epithelial breast cells, in the presence of Zn(II)PPIX‐loaded NMOFs gated with VEGF aptamer ‐modified DNA tetrahedra. Panel II‐Cell viability of (i) MDA‐MB‐231 breast cancer cells and (ii) MCF‐10A epithelial breast cells treated with the Zn(II)PPIX‐loaded NMOFs gated with the VEGF‐responsive DNA tetrahedra upon photodynamic treatment. (A)–(C) Reproduced with permission.^[^ ^135^ ^]^ Copyright 2021, Elsevier. (D)–(G) Reproduced with permission.^[^ ^136^ ^]^ Copyright 2018, Wiley. (H)–(J) Reproduced under terms of a Creative Commons Attribution‐Non Commercial 3.0 Unported Licence.^[^ ^137^ ^]^ Copyright 2021, RSC.

**Figure** **4**A exemplifies the application of a hybrid composite consisting of tetracarboxyphenyl‐meso‐porphyrin (TCPP)/G‐quadruplex‐modified Au NPs for the photodynamic treatment of HeLa cancer cells.^[^ ^135^ ^]^ The Au NPs were modified with thiolated AS1411 aptamer known to form a G‐quadruplex that binds to the TCPP by cooperative electrostatic and π‐π interactions, resulting in the photosensitizer for photosensitized generation of ^1^O_2_, the toxic ROS toward the cancer cells. *In vitro* cell experiments (Figure 4B) demonstrated the photodynamic effect of ^1^O_2_ on the viability of the HeLa cancer cells. While the light‐activated (λ_ex_ = 650 nm) TCPP/G‐quadruplex‐modified Au NPs revealed effective ROS cytotoxic effect on the cell viability, reflected by a 90% cell death after 20 min illumination, the control systems consisting of non‐modified particles or modified composite nanoparticles in the dark revealed minute effects on the cells. Also, HeLa tumors elicited in xenograft mice were subjected to intrathecal (IT) injection of the TCPP/G‐quadruplex‐modified Au NPs and subjected to photodynamic treatment, Figure 4C. While the tumor growth in the illuminated mice treated with the TCPP/G‐quadruplex‐modified Au NPs was fully blocked along a time period of 14 days, a 4‐fold growth of the tumors was observed upon treatment of the mice with the control, non‐ROS generating systems.

The combined photodynamic, chemodynamic and photothermal treatment of the cancer cells is exemplified in Figure 4D.^[^ ^136^ ^]^ Au NPs were modified with thiolated G‐rich strand (1), hybridized with complementary strand (2). The strand (1) self‐assembled in the presence of K^+^ into G‐quadruplex structure, enabling the binding of Zn(II) phthalocyanine (ZnPc) as functional photosensitizer for the photoinduced generation of reactive ^1^O_2_ species. The duplex domains between (1)/(2) allowed the intercalation of the DOX, chemotherapeutic drug. Moreover, the strand (1) was pre‐engineered to include the sequence “w” that consists of a cytosine‐rich sequence corresponding to an i‐motif subunit. The visible light illumination (λ_ex_ = 660 nm) of the hybrid ZnPc‐functionalized G‐quadruplex system leads to the formation of the ^1^O_2_ cytotoxic agents. The permeation of the hybrid particles into the acidic cancer cells leads, however, to the separation of the strands (1) through the formation of interparticle i‐motif bridged Au NPs aggregates. While the pH‐stimulated separation of the (1)/(2) duplexes leads to the release of the chemotherapeutic DOX drug as co‐cytotoxic agent toward the cancer cells, the accompanying aggregation of the Au NPs leads to the formation of interparticle plasmon excitons. These allow, then, the NIR thermoplasmonic heating of the cells, as a further inducer of cell death. That is, the hybrid DNA/NPs exhibits cooperative photodynamic/chemotherapeutic/photothermal functionalities affecting the cell apoptosis. Figure 4E demonstrates the different functions of the components associated with the ZnPc/G‐quadruplex/Au NPs hybrid. The capacity of the hybrid system to generate ^1^O_2_ species under light (λ_ex_ = 660 nm) is presented in Panel I. The capacity of the hybrid to be separated at pH 5.0 and release the chemotherapeutic DOX drug is evidenced in Panel II. The photothermal effect of the aggregated Au NPs using NIR excitation (λ_ex_ = 808 nm) is evidenced in Panel III, and the pH‐stimulated aggregation of the Au NPs is displayed in Panel IV, followed by TEM. The *in vitro* cooperative effect of the hybrid ZnPc/G‐quadruplex‐functionalized Au NPs on MDA‐MB‐231 breast cancer cells is presented in Figure 4F. While the irradiation of the cells with the two light sources (660 nm for ZnPc excitation, 808 nm for Au aggregates irradiation) in the absence of the hybrid composite has no effect on the cell viability, the stepwise treatment of the cells with the composite in the dark (releasing only DOX) leads to ca. 50% cell death, the cooperative photodynamic/DOX release leads to ca. 70% cell death and the cooperative DOX release/NIR selective irradiation of the resulting Au aggregates leads also to ca. 70% cell death, the cooperative effect of photodynamic/chemotherapeutic/photothermal treatment, lead to ca. 85% cell death. The in vivo experiments involving IT treatment of xenograft mice carrying MDA‐MB‐231 tumors validated the cellular experiment and demonstrated the effective cooperative photodynamic/chemotherapeutic/photothermal effects on the inhibition of the tumor growth, Figure 4G.

Nanoparticles loaded with photosensitizers and modified with DNA constituents may be engineered into functional nanostructures that emerge, in the presence of intracellular cancer cell biomarkers, into active photodynamically active configurations. This is exemplified in Figure 4H with the loading of nucleic acid (3)‐modified UiO‐66 NMOFs with Zn(II) PPIX photosensitizer.^[^ ^137^ ^]^ The loaded NMOFs was caged with DNA tetrahedra nanostructures, modified at one corner with a single‐strand nucleic acid (3’) to form the duplex (3)/(3’)‐bridged tetrahedra/NMOFs hybrid. DNA tetrahedra nanostructure exhibits superior cell permeation capacities, and thus, the DNA‐modified NMOFs provide effective cell permeation vehicles. The strand (3’) was engineered, however, to include the anti‐VEGF aptamer sequence, known to have a G‐quadruplex configuration, upon forming the VEGF/aptamer complex. Realizing the VEGF is overexpressed in cancer cells, the cancer cell permeated tetrahedra/NMOFs conjugate are unlocked by VEGF forming the VEGF/aptamer‐tetrahedra constituents and the concomitant intracellular release of the Zn(II)PPIX photosensitizer. The porphyrinic Zn(II)PPIX associates then with the G‐quadruplex/VEGF aptamer‐tetrahedra constituents generating a superior Zn(II)PPIX/G‐quadruplex photosensitizer for the effective photosensitized generation of toxic ROS toward the cancer cells. Figure 4I demonstrates the effective in vitro uncaging of the loaded NMOFs by VEGF and the release of the Zn(II) PPIX load, Panel I. The effective generation of ROS by the resulting Zn(II) PPIX/G‐quadruplex constituents is presented in Panel II. Indeed, in vitro cell experiments demonstrated the effective photoinduced formation of ROS species upon illumination of MDA‐MB‐231 breast cancer cells treated with the DNA tetrahedra‐modified NMOFs, Figure 4J, Panel I, and effective cancer cell death upon illumination was presented, Panel II.

### 3.2 DNAzyme‐Modified Nanoparticles for Catalytic Cancer Therapy

**Figure 5:**
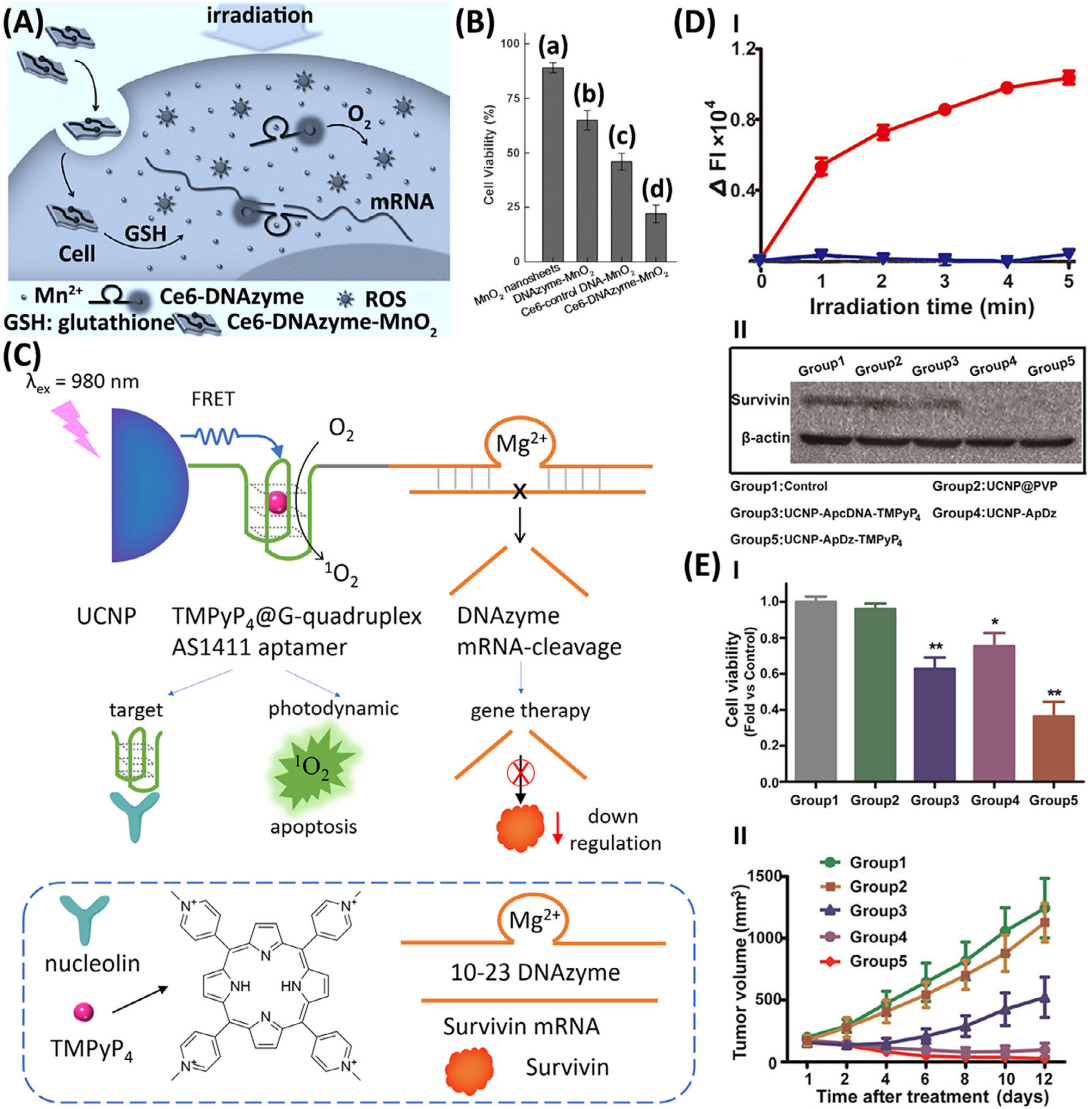
A) Schematic application of MnO_2_ nanoparticles modified with Mn^2+^‐dependent DNAzyme strand functionalized with chlorin e6 (Ce6) as hybrid conjugates for the cooperative gene silencing and photodynamic treatment of MCF‐7 cancer cells. The GSH‐stimulated reduction of MnO_2_ to Mn^2+^ ions activates the Mn^2+^‐dependent DNAzyme cleaving EGR‐1 mRNA and the Ce6 photosensitizer cooperatively activates the photodynamic generation of cytotoxic ^1^O_2_ species. B) MCF‐7 cells viability in the presence of: (a) non‐modified MnO_2_ particles, (b) MnO_2_ particles modified with the DNAzyme strand in the dark, (c) MnO_2_ particles modified a random strand functionalized with the photosensitizer, under light (only photodynamic effect), (d) the DNAzyme‐modified MnO_2_ particles under light demonstrating gene silencing and photodynamic activities. C) Schematic upconversion nanoparticles (UCNPs) stimulated cooperative photodynamic and DNAzyme gene silencing treatment of cancer cells. UCNPs are functionalized with RCA chains composed of tetramethyl pyridinium porphyrin, TMPyP_4_‐loaded G‐quadruplex (G4) for photodynamic therapy and structurally engineered Mg^2+^‐ion‐dependent DNAzyme units cleaving the survivin mRNA. D) Panel I‐Temporal formation of ^1^O_2_ by the TMPyP_4_‐loaded G4 chains associated with the UCNPs: (i) upon irradiation (λ_ex_ = 980 nm), (ii) in the park. Panel II‐Western blot images corresponding to the cleavage of the survivin mRNA and expressed survivin (upper lane), and control mRNA expression of β‐actin in MCF‐7 cells treated with: (1) buffer solution, (2) UCNPs, (3) UCNPs modified with a random DNAzyme, (4) UCNPs modified with the DNAzyme/G4 lacking TMPyP_4_, (5) UCNPs modified with the DNAzyme and TMPyP_4_‐loaded G4. E) Panel I‐MCF‐7 cell viability and Panel II‐Inhibition of MCF‐7 tumor growth upon treated with conditions (1)–(5) outlined in (D) Panel II. (A),(B) Reproduced with permission.^[^ ^138^ ^]^ Copyright 2015, Wiley. (C)–(E) Reproduced with permission.^[^ ^139^ ^]^ Copyright 2020, ACS.

The catalytic function encoded in oligonucleotides (DNAzymes) was employed for gene silencing and cancer therapy through DNAzyme/particle conjugates.^[^ ^138^ ^]^ This is exemplified in **Figure** **5**A with the cancer cell‐dictated evolution of a Mn^2+^‐dependent DNAzyme that silences the mRNA associated with the human early growth response‐1 (EGR‐1) protein, thereby inducing apoptosis of the cancer cells. Moreover, the DNAzyme/particle conjugate is functionalized with a photosensitizer leading to the cooperative photodynamic and gene therapy for cancer cells. MnO_2_ particles were used as carriers of the chlorin e6 (Ce6)‐labeled 10–23 DNAzyme sequence. The mechanism of the cooperative operation of the Ce6‐DNAzyme‐modified MnO_2_ particle is displayed in Figure 5A. The cell permeated Ce6‐DNAzyme‐modified particles react with glutathione, GSH, overexpressed in cancer cells, resulting in the formation of Mn^2+^ and the release of the Mn^2+^‐DNAzyme framework into the cytoplasm. The resulting cleavage of the mRNA associated with the translation of EGR‐1 leads to gene silenced apoptosis of the cells. In addition, the release of the porphyrinic Ce6‐labeled DNAzyme leads, upon illumination, to the formation of cytotoxic ^1^O_2_ species, leading to the photodynamic degradation of the cancer cells. Figure 5B reveals the cooperative therapeutic effects of the hybrid Ce6‐DNAzyme/MnO_2_ on MCF‐7 breast cancer cells. While in the dark only the GSH driven Mn^2+^‐DNAzyme gene silencing path is operated, leading to 45% MCF‐7 breast cancer cell death, the cooperative photodynamic and gene silencing activation of the hybrid Ce6‐DNAzyme/MnO_2_ particles lead to ca. 80% cancer cell death.

The cooperative porphyrinic/G‐quadruplex photodynamic and DNAzyme gene silencing treatment of cancer cells is displayed in Figure 5C.^[^ ^139^ ^]^ Upconversion nanoparticles (UCNPs) were functionalized with rolling circle amplified (RCA) chains composed of G‐quadruplex (AS1411 aptamer) and 10–23 DNAzyme subunits. The tetramethyl pyridinium porphyrin, TMPyP_4_, was bound by cooperative electrostatic and π‐π interaction to the G‐quadruplex unit. While the TMPyP_4_ photosensitizer/G‐quadruplex unit acted as the photodynamic agent, the 10–23 DNAzyme sequence was engineered to silence the mRNA associated with the survivin, leading to the apoptosis of the cells. The UCNPs function as NIR absorbent that upon excitation (λ_ex_ = 980 nm) transfer energy to the porphyrinic agents leading to their photoactivation toward formation of the cytotoxic ^1^O_2_ agents. The effective *in vitro* generation of ^1^O_2_ by the NIR‐illumination of the TMPyP_4_/G‐quadruplex functionalized UCNPs is displayed in Figure 5D, Panel I, and the effective gene silencing of the mRNA translated survivin in MCF‐7 breast cancer cells treated in the dark with the TMPyP_4_/G‐quadruplex/DNAzyme‐modified UCNPs or UCNPs modified with TMPyP_4_‐free G‐quadruplex/DNAzyme RCA chains, is presented in Panel II. The *in vitro* cooperative photodynamic and gene silencing treatment of MCF‐7 cancer cells with the TMPyP_4_/G‐quadruplex/DNAzyme UCNPs is presented in Figure 5E, Panel I. While the TMPyP_4_‐free G‐quadruplex/DNAzyme particle, resulting in only the mRNA silencing effect, revealed only 30% cell death, the NIR‐light activated UCNPs modified with TMPyP_4_/G‐quadruplex/DNAzyme chains, exhibiting the cooperative photodynamic and gene silencing activities, revealed 65% cell death. These results are complemented by in vivo experiments probing the growth inhibition of MCF‐7 tumors in xenograft mice, upon IT injection TMPyP_4_/G‐quadruplex/DNAzyme‐modified UCNPs, Figure 5E, Panel II. While treatment of the tumors in the dark, executing only the gene silencing effect, the tumor growth was inhibited by ca. 60%, the light‐treated samples exposed to the photodynamic and cooperative gene silencing pathway revealed almost 100% growth inhibition.

## 4 Stimuli‐Responsive DNA‐Gated Nanoparticles for Cancer Therapy

Diverse, highly‐porous inorganic nanoparticles, such as SiO_2_, ZrO_2_, TiO_2_ or NMOFs have been employed as drug carriers. The caging of the carriers by stimuli‐responsive nucleic acids and their unlocking by cellular biomarkers, such as miRNA or cell‐specific environment agents including metal ions or pH, and the accompanying intracellular controlled drug release provide versatile means for therapeutic application, particularly cancer cell treatment. Many reviews addressing this topic were reported.^[^ ^81^, ^82^, ^83^ ^]^ In the present section, the use of stimuli‐responsive nucleic acid‐functionalized NMOFs for chemotherapeutic cancer cell treatment will be exemplified.

**Figure 6:**
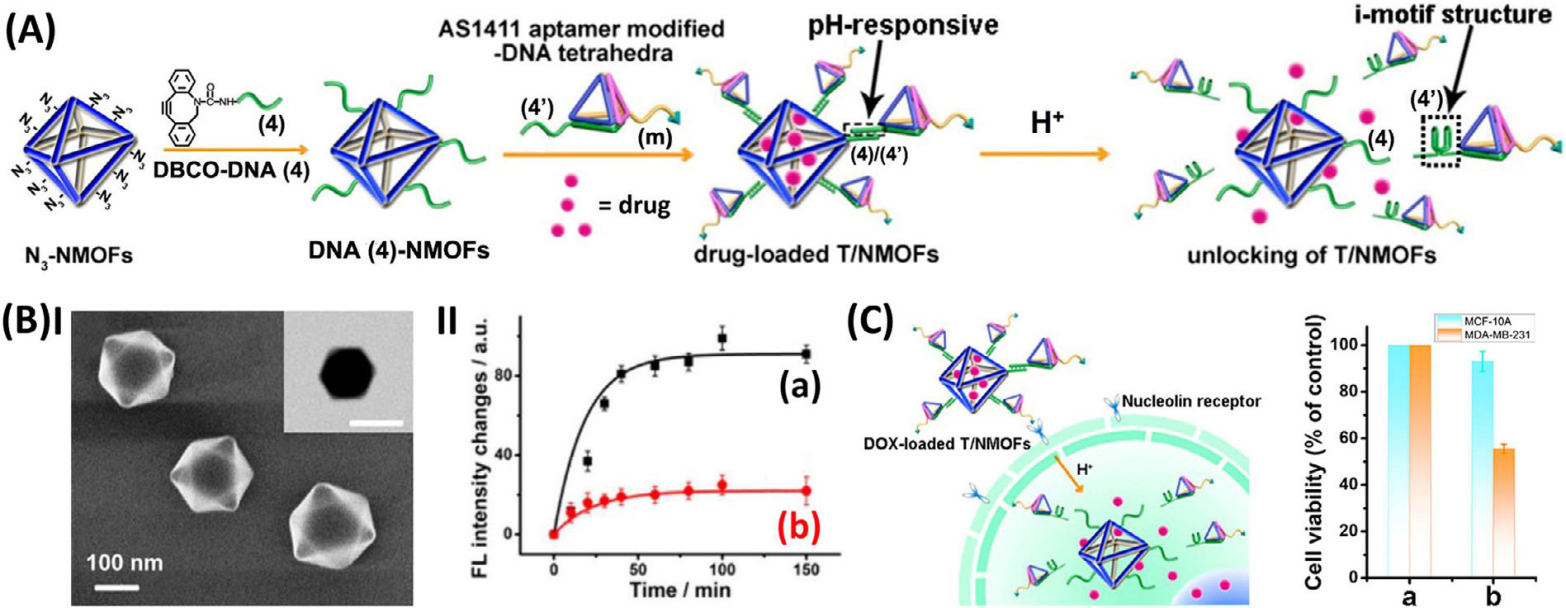
A) Schematic assemble of DNA tetrahedra‐modified, pH‐responsive, doxorubicin (DOX)‐loaded UiO‐66 NMOFs and their pH‐stimulated uncaging release of the drug. B) Panel I‐SEM and TEM images of the NMOFs. Panel II‐(a) pH‐triggered time dependent release of DOX from the NMOFs, pH 5.5, (b) inhibited release of the DOX from the NMOFs at pH 7.2. C) Cytotoxicity of the DOX‐loaded NMOFs toward MDA‐MB‐21 breast cancer cells and control MCF‐10 epithelial breast cells upon: (a) non‐treated control, (b) treated with drug‐loaded NMOFs, 30 µg mL^−1^, for 5 days. (A)–(C) Reproduced with permission.^[^ ^140^ ^]^ Copyright 2021, ACS.

The preparation and operating mechanism of DNA‐modified pH‐responsive UiO‐66 NMOFs as hybrid conjugate for chemotherapeutic treatment of cancer cells is displayed in **Figure** **6**A.^[^ ^140^ ^]^ The design of the hybrid pH‐responsive drug carrier is based on the fact that pH environment of cancer cells is acidic and differs from the pH of normal cells, suggesting that pH‐responsive nanocarriers could provide selective controlled drug release in the cancerous cells. Azide‐functionalized UiO‐66 NMOFs were modified with nucleic acid anchoring strand (4), loaded with the chemotherapeutic drug, DOX, and caged by hybridizing DNA tetrahedra, functionalized at their corners with complementary tether (4’) and the AS1411 aptamer tether “m”. The strand (4’) bridging the tetrahedra units to the NMOFs includes a cytosine‐rich, pH‐responsive sequence. While the NMOFs, Figure 6B, Panel I, provide a porous framework for loading DOX drug, the tetrahedra unit provides effective cell permeation of the carrier and the tethered aptamer introduces a cell targeting capacity. At acid pH, present in cancer cells, the bridging unit (4’) reconfigures into an i‐motif leading to the separation of the tetrahedra unit, resulting in the unlocking of the carrier and the release of the loaded drug, Figure 6B, Panel II. Treatment of MDA‐MB‐231 breast cancer cell with the pH‐responsive DNA tetrahedra/NMOFs hybrid framework demonstrated selective cytotoxicity toward the cancer cells, as compared to normal epithelial MCF‐10A breast cells, Figure 6C. The hybrid conjugates induced ca. 45% cell death of the cancer cells, whereas the normal cells were not affected. The selectivity was attributed to the effective targeted, DNA tetrahedra‐assisted permeation of the particles into the cancer cells, leading to the subsequent pH‐stimulated unloading of the frameworks in the cancer cells.

**Figure 7:**
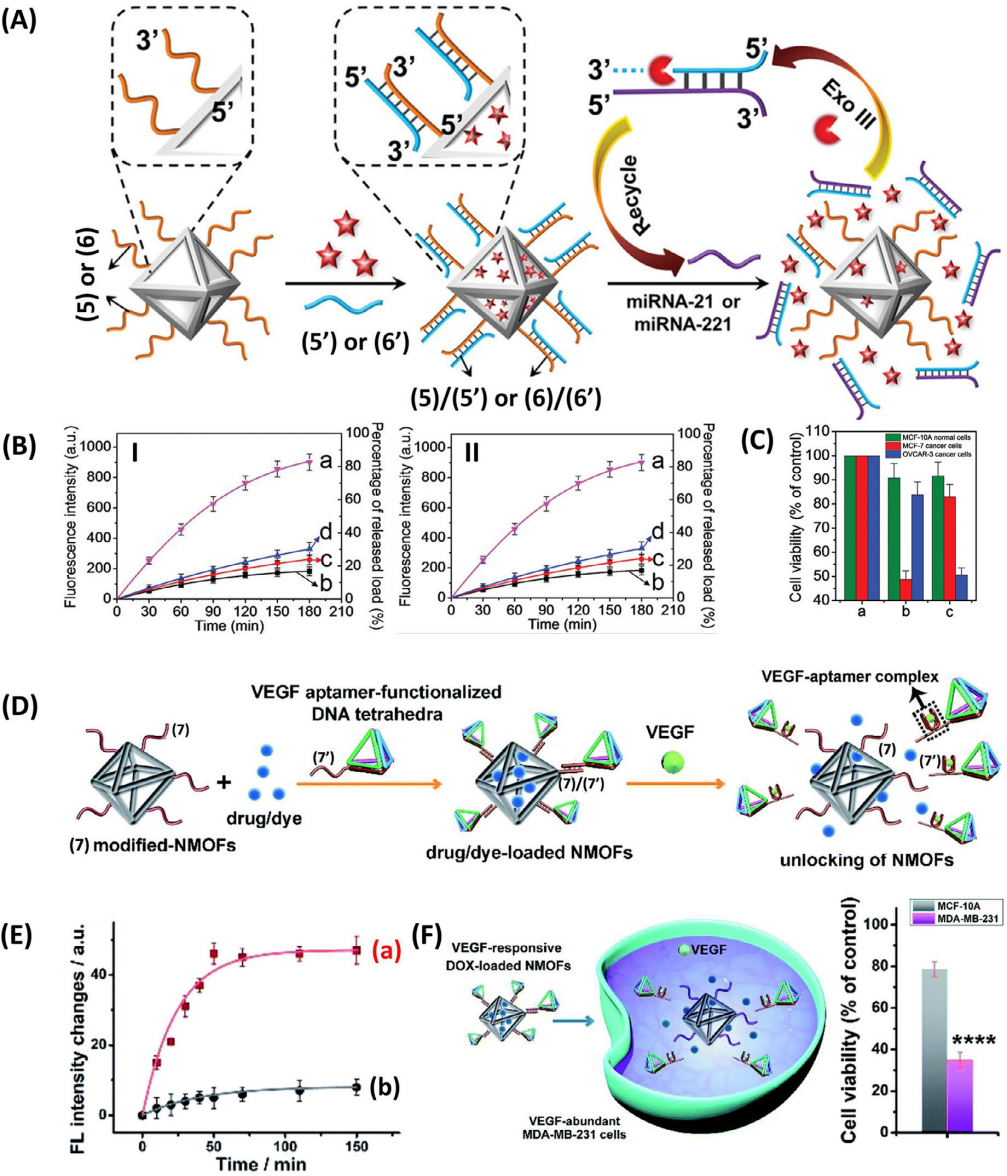
A) Schematic assembly of DOX‐loaded miRNA‐responsive UiO‐68 NMOFs and triggered miRNA‐responsive release of the loads through the Exo‐III regeneration of miRNA biomarkers. B) Panel I‐Release of the DOX‐loaded NMOFs caged by the (5)/(5’)‐miRNA‐21 responsive NMOFs: (a) in the presence of the miRNA‐21 and Exo‐III, (b) in the absence of added miRNA‐21, (c) in the presence of foreign miRNA‐221, (d) in the presence of miRNA‐21 and the absence of added Exo‐III. Panel II‐Release of the DOX‐loaded NMOFs caged by the (6)/(6’)‐miRNA‐221 responsive NMOFs: (a) in the presence of the miRNA‐221 and Exo‐III, (b) in the absence of added miRNA‐221, (c) in the presence of foreign miRNA‐21, (d) in the presence of miRNA‐221 and the absence of added Exo‐III. C) Selective cytotoxicity of the miRNA‐responsive DOX‐loaded NMOFs toward MCF‐7 breast cancer cells (overexpress miRNA‐21) and OVCAR‐3 ovarian cancer cells (overexpress miRNA‐221) in comparison to non‐cancerous epithelial MCF‐10A breast cells: (a) non treated cells, (b) treatment of the cells with the (5)/(5’)‐miRNA‐21 responsive NMOFs, (c) treatment of the cells with the (6)/(6’)‐miRNA‐21 responsive NMOFs. D) Assembly of DNA tetrahedra‐modified DOX‐loaded VEGF‐responsive UiO‐66 NMOFs and VEGF‐induced uncaging and drug release in the presence of overexpressed VEGF in cancer cells (uncaging process via formation of the respective VEGF‐aptamer complex). E) Time‐dependent release of the DOX from the VEGF‐responsive NMOFs: (a) in the presence of added VEGF, 2 µM, (b) in the absence of added VEGF. F) Cytotoxicity of the VEGF‐responsive, DOX‐loaded UiO‐66 NMOFs toward MDA‐MB‐231 breast cancer cells and normal MCF‐10A breast epithelial cells. (A)–(C) Reproduced with permission.^[^ ^141^ ^]^ Copyright 2019, Wiley. (D)–(F) Reproduced under terms of a Creative Commons Attribution‐Non Commercial 3.0 Unported Licence.^[^ ^137^ ^]^ Copyright 2021, RSC.

Beyond unloading of the drug‐loaded nucleic acid caged NMOFs by environmental conditions present in the cancer cells, specific cancer cell biomarkers can be employed to selective release the chemotherapeutic drug in cancer cells. This is exemplified in **Figure** **7**A using miRNA biomarkers.^[^ ^141^ ^]^ miRNA is short, single‐stranded, non‐coding RNA overexpressed in different kinds of cancer cells. Figure 7A depicts the principle to unlock the drug loaded UiO‐68 NMOFs by one of two miRNAs, miRNA‐21 (overexpressed in MCF‐7 breast cancer cells) or miRNA‐221 (overexpressed in ovarian OVCAR‐3 cancer cells). The UiO‐68 NMOFs were modified with nucleic acid, (5) or (6), loaded with the DOX chemotherapeutic drug and caged with the complementary nucleic acid, (5’) or (6’), Panel I. The caging strand (5’) or (6’) was pre‐engineered, however, to include the recognition sequence to be displaced by the respective miRNA‐21 or miRNA‐221. The mechanism to unlock and release of the chemotherapeutic drug is, also, displayed in Figure 7A. In the presence of miRNA‐21 or miRNA‐221, the caging strand (5’) or (6’) is displaced forming (5’)/miRNA‐21 or (6’)/miRNA‐221 duplex. The typical concentration of the miRNA biomarkers is relatively low, and thus the uncaging process and release of the drug are inefficient. Accordingly, an auxiliary or cellular mechanism to enhance the uncaging process is needed. This is achieved by the implementation of an auxiliary (or cellular) exonuclease, that cleaves the DNA constituents in the (5’)/miRNA‐21 or (6’)/miRNA‐221 duplex, resulting the regeneration of the miRNA biomarkers and the enhanced uncaging of the drug‐loaded NMOF carriers. Figure 7B depicts the Exo III‐assisted release of DOX from the (5’)‐caged NMOFs by miNRA‐21, Panel I, whereas the miRNA‐221 induced, Exo III‐assisted release DOX from the (6’)‐caged NMOFs, is displayed in Panel II. The selective, miRNA‐dictated cytotoxicity of the miRNA‐responsive caged NMOFs is displayed in Figure 7C. Treatment of the MCF‐7 breast cancer cells (containing overexpressed miRNA‐21) with (5’)‐caged NMOFs reveals ca. 55% cell death while the normal epithelial MCF‐10A breast cells, or the OVCAR‐3 cancer cells are almost unaffected. In contrast, treatment of the OVCAR‐3 cells (containing overexpressed miRNA‐221) with the (6’)‐caged, miRNA‐221‐responsive NMOFs leads to ca. 50% cell death of the OVCAR‐3 cells, while the MCF‐10A or MCF‐7 cells are almost not affected. These results demonstrated the cytotoxic selectivity stimulated by the miRNA‐responsive NMOFs. It should be noted that the permeation of the miRNA‐responsive MOFs carriers into the MCF‐7 or OVCAR‐3 cells is quite similar^[^ ^141^ ^]^ and thus, the observed selective cytotoxicity is dictated by the miRNA biomarkers.

Besides miRNA as cancer cell biomarkers, overexpressed proteins, such as VEGF, provide similar biomarker for cancer cells. Accordingly, aptamer against protein biomarkers was employed as caging strands locking drug loaded NMOFs by the formation of protein biomarker/aptamer complex. This is exemplified in Figure 7D with the development of DOX‐loaded NMOFs gated by VEGF aptamer lock for the chemotherapeutic treatment of cancer cells.^[^ ^137^ ^]^ Nucleic acid (7)‐functionalized UiO‐66 NMOFs were loaded with the DOX drug and the loaded particles were caged by DNA‐tetrahedra functionalized at one corner with the single strand tether (7’) complementary to the strand (7) associated with the NMOFs carriers. While the DNA‐tetrahedra units provide nano‐vehicles for assisted permeation of the NMOFs into cells, the tether (7’) was engineered to include the anti‐VEGF aptamer sequence. Accordingly, in the presence of VEGF, the caging units are unlocked through generation of the aptamer/VEGF complex, resulting in the release of the DOX load. Figure 7E depicts the time‐dependent VEGF‐induced release of the DOX from the carrier. The selective cytotoxicity effect of the drug‐loaded NMOFs on MDA‐MB‐231 breast cancer cells is displayed in Figure 7F. Treatment of the cancer cells with the caged drug carrier resulted in ca. 70% cell death, while control MCF‐10A epithelial breast cells revealed only 20% cell death under similar condition. The resulting selectivity was attributed to the enhanced tetrahedra assisted permeation of the carrier into the cancer cells and to the enhanced unlocking of the carriers in the cancer cells by overexpressed VEGF.

## 5 Nucleic Acid/Nanoparticle Hybrids for Gene Therapy

Short‐strand oligonucleotides silencing genes find growing interest for gene therapy. These include double‐stranded RNA silencing gene, siRNA,^[^ ^142^, ^143^ ^]^ or antisense oligonucleotide,^[^ ^144^, ^145^ ^]^ ASO, consisting of short, synthetic single‐stranded DNA silencing mRNA or other RNA constituents controlling mRNA expression, such as Pre‐mRNA, miRNA or IncRNA. The implementation of these short‐strand nucleic acids for therapeutic applications suffers, however, from common limitations associated with limited permeation and delivery into cells and their limited stability toward environmental degrading enzymes in the circulative bloodstream or intracellular biocatalysts. Accordingly, substantial efforts are directed to integrate the siRNA or ASO in functional assemblies stabilizing oligonucleotide constituents against degradation and concomitantly assisting their targeted permeation into cells. Diverse micro/nano‐structures were employed as stabilizing vehicles for siRNA and ASO, including polymeric nanoparticles,^[^ ^146^ ^]^ liposomes,^[^ ^147^ ^]^ dendrimers,^[^ ^148^ ^]^ lipid nanoparticles,^[^ ^149^ ^]^ DNA nanostructures,^[^ ^150^ ^]^ metal oxide particles^[^ ^151^ ^]^ and more.^[^ ^152^, ^153^ ^]^ Indeed, spherical nucleic acids (SNAs) consisting of micellar oligonucleotides, liposomes, nucleic acid‐loaded dendrimers or polymers, and nucleic acid‐modified nanoparticles, are broadly used as hybrid structures for carrying siRNA or ASO constituents for gene therapy.^[^ ^89^, ^90^ ^]^ Not surprisingly, the high‐surface‐area and surface functionalities associated with nanoparticles, together with their catalytic, photocatalytic and plasmonic properties, attract growing interest as functional carriers for siRNA and ASO. The conjugation of siRNA and ASO to nanoparticles and the application of the hybrid as vehicles to enhance gene therapy will be exemplified in the forthcoming section.

### 5.1 siRNA/Nanoparticle Conjugates for Gene Therapy

siRNA is typically 20–25 base‐pair RNA duplex that binds to the intracellular protein forming RNA‐induced silencing complex (RISC), where the duplex is separated into a guide silencing strand/RISC complex that binds to the complementary mRNA region being cleaved by the complex. Degradation of the mRNA leads to subsequent inhibition of protein translation. The RISC provides versatile key mechanism for gene silencing and gene therapy. The major drawbacks in siRNA gene therapy are, however, limited cellular uptake and stability of the siRNA agent. Conjugation of siRNA to diverse carriers such as DNA carriers, e.g., tetrahedra,^[^ ^154^ ^]^ origami frameworks,^[^ ^155^ ^]^ nanogels,^[^ ^156^ ^]^ polymers^[^ ^157^ ^]^ and carriers such as liposomes^[^ ^158^ ^]^ or porous materials,^[^ ^159^ ^]^ was employed to overcome these difficulties. Substantial recent interest included the application of nanoparticles as carriers of siRNA. Surface functionalization of the particles assists the targeted permeation of the siRNA into the cancer cells, and enhances the stability toward enzymatic degradation of the siRNA. Moreover, by appropriate engineering of the carrier, cooperative gene therapy and photodynamic/photothermal therapeutics by the siRNA/particle conjugates may be designed.

**Figure 8:**
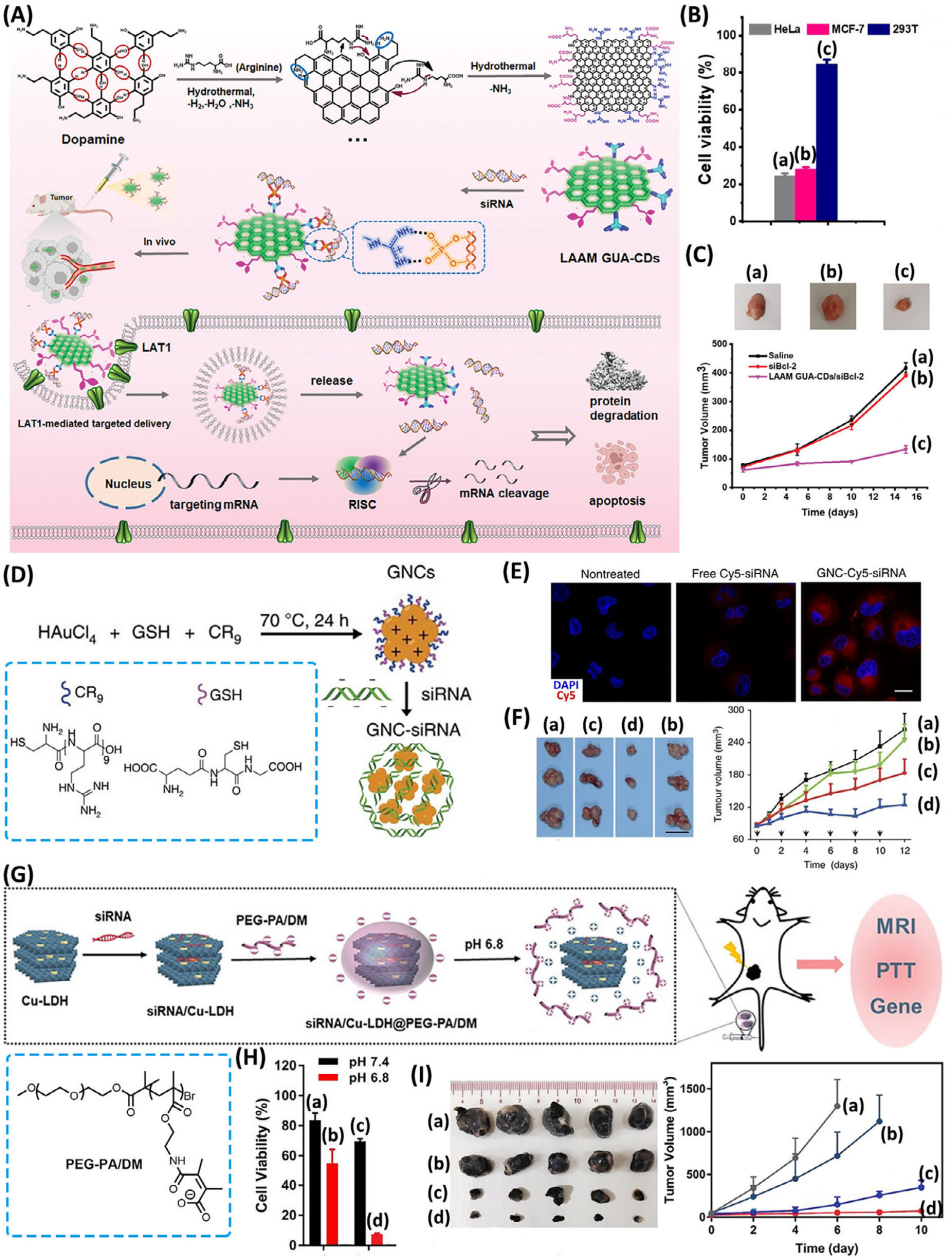
A) Hydrothermal synthesis of carbon dots (C‐dots) modified with COOH/NH_2_ residues and positively charged guanidinium units anchoring siRNAs inhibiting the expression of the B‐cell lymphoma 2 (Bcl‐2) gene in HeLa or MCF‐7 breast cancer cells. B) Viability of different cell lines treated with the Bcl‐2 siRNA‐modified C‐dots: (a) HeLa cells, (b) MCF‐7 cells, (c) 293T normal epithelial cells. C) Time dependent HeLa tumor growth treated with: (a) saline only, (b) free siRNA, (c) siRNA‐modified C‐dots. D) Synthesis of glutathione (GSH) and oligoarginine‐modified Au nanoclusters (NCs) functionalized with siRNA silencing the gene of the nerve growth factor (NGF) overexpressed in pancreatic cancer cells (Panc‐1). E) Confocal fluorescence microscopy images corresponding to the uptake of the siRNA‐modified Au NCs as compared to the uptake of the free siRNA units. F) Time‐dependent growth of the Panc‐1 tumors in xenograft mice upon treatment with: (a) saline, (b) random RNAs modified Au NCs, (c) free siRNA silencing the NGF gene, (d) Au NCs modified with siRNA silencing the NGF gene. G) Synthesis of siRNAs‐loaded Cu^2+^‐modified layered double Mg/Al hydroxides (Cu^2+^‐LDH), stabilized with a pH‐responsive charge reversed copolymer coating PEG‐PA/DM, for cooperative gene silencing/photothermal treatment of B16F0 melanoma cancer cells. H) Viability of B16F0 cells upon treatment with siRNA‐modified Cu^2+^‐LDH at: (a) pH 7.4, in the dark, (b) pH 6.8, in the dark, (c) pH 7.4, 808 nm laser irradiation for 300 s (1 W cm^−2^), (d) pH 6.8, 808 nm laser irradiation for 300 s (1 W cm^−2^). I) Time‐dependent growth of the B16F0 tumors in xenograft mice upon treatment with: (a) PBS, (b) siRNA‐modified Cu^2+^‐LDH in the dark, (c) Cu^2+^‐LDH irradiated with 808 nm laser, (d) siRNA‐modified Cu^2+^‐LDH irradiated with 808 nm laser. (A)‐(C) Reproduced with permission.^[^ ^160^ ^]^ Copyright 2023, Wiley. (D)–(F) Reproduced under terms of a Creative Commons Attribution 4.0 International License.^[^ ^161^ ^]^ Copyright 2017, Springer Nature. (G)–(I) Reproduced with permission.^[^ ^162^ ^]^ Copyright 2020, Wiley.

**Figure** **8**A exemplifies the application of carbon dots (C‐dots)‐functionalized with amino acid COOH/NH_2_ and positively charged guanidinium units, prepared by hydrothermal treatment of dopamine and arginine, as functional nanocarrier of siRNA.^[^ ^160^ ^]^ While the amino acid residues target the particles to the large neutral amino acid transporter 1 (LAT1) receptor sites overexpressed on cancer cell membranes, thus facilitating the permeation of the C‐dots carrier to the cancer cells, the guanidinium units associated with the carrier bind electrostatically the phosphate residues associated with the siRNAs, thereby facilitating the transport of siRNAs and inhibition of cellular mRNA. Accordingly, HeLa or MCF‐7 breast cancer cells treated with the B‐cell lymphoma 2 (Bcl‐2) gene siRNA‐loaded C‐dots, resulting in the inhibition of Bcl‐2 protein and the apoptosis of the cancer cells, Figure 8B. Evidently, ca. 80% cell death of the cancer cells, containing overexpressed LAT1 and downregulated Bcl‐2 protein, was observed, while normal 293T cells lacking the LAT1 receptor were almost unaffected. Moreover, in vivo experiments using HeLa tumor bearing mice demonstrated the gene therapeutic advantages of the Bcl‐2 siRNA‐loaded carrier over control systems, Figure 8C. While treatment of the tumors with the pure siRNA agent did not show any significant effect on the tumor growth, presumably due to the inefficient permeation or the insufficient stability, impressive inhibition of the tumor growth was observed upon treatment of the mice with the siRNA‐loaded C‐dots.

In a further example, Figure 8D, Au nanoclusters (NCs) modified with GSH as cell‐permeating agent and thiolated oligoarginine siRNA‐binding sites were used as carrier of siRNA silencing the gene expressing the nerve growth factor (NGF) overexpressed in pancreatic cancer cells, Panc‐1.^[^ ^161^ ^]^ Electrostatic binding of the siRNA to the oligoarginine units facilitated the permeation of the siRNA into the Panc‐1 cells, Figure 8E. The Au NCs carrier exhibited enhanced delivery of the siRNA into the subcutaneous Panc‐1 tumors elicited in xenograft mice, as compared to inefficient delivery of bare siRNA into the tumors, revealing substantial improved inhibition of the tumor growth by the siRNA‐modified Au NCs carrier, Figure 8F. While a foreign siRNA bound to the carrier did not affect the growth of the tumors, the bare siRNA inhibited the growth of the tumors by ca. 50% and the siRNA‐modified Au NCs inhibited the tumor growth by 90%.

In a further example, siRNA‐loaded Cu^2+^‐ modified layered double hydroxide nanoparticles, Cu^2+^‐LDH, were employed for the cooperative gene therapy/photothermal therapy of B16F0 melanoma cancer cells, Figure 8G.^[^ ^162^ ^]^ The positively charged Cu^2+^‐LDH nanoparticles were loaded with the siRNA silencing the Bcl‐2 gene overexpressed in B16F0 cells, and the particles were stabilized by a dimethyl maleic acid‐aminoethylacrylo‐PEG negatively charged coating (PEG‐PA/DM). The acid, pH 6.8, in the vicinity of the cancer cells, desorbed the coating layer, resulting the enhanced permeation of the positively charged siRNA‐loaded nanoparticles into the cancer cells. The intracellular release of the siRNA resulted in the Bcl‐2 silencing. Moreover, the light‐induced excitation (λ_ex_ = 808 nm) of the Cu^2+^‐LDH nanoparticles resulted in the photothermal heating by the particles leading to a phototherapeutic effect and eventually to enhanced dissociative release of the siRNA, Figure 8G. In vitro cell experiments demonstrated the cooperative gene and photothermal therapeutic effects on the cell viability, Figure 8H. While in the absence of light, the cell subjected to the siRNA‐loaded Cu^2+^‐LDH revealed a cell death of 45%, the cells subjected to the cooperative siRNA and photothermal effect showed a cell death corresponding to 90%. The cooperative siRNA silencing and photothermal effects of the Cu^2+^‐LDH nanoparticles were also demonstrated by in vivo experiment using B16F0 tumor‐bearing xenograft mice, Figure 8I. While the mice treated with the bare siRNA revealed 20% tumor growth inhibition, the tumor subjected to siRNA‐free Cu^2+^‐LDH demonstrated 70% growth inhibition, due to the photothermal effect, and the cooperative gene silencing and photothermal effect led to > 90% tumor growth inhibition.

### 5.2 Antisense/Nanoparticle Conjugates for Gene Therapy

Antisense oligonucleotides (ASO) are short, synthetic single‐stranded oligonucleotides that bind to cellular mRNA or to precursor RNAs regulating the expression of mRNA, such as miRNA, rRNA, tRNA and more, thereby silencing the mRNA and the downstream translation of proteins dictating cell functions, such as apoptosis or proliferation. Indeed, ASO find growing interest as gene regulating agents treating diverse diseases, such as cancer, cardiovascular and metabolic disorders and more. As stated, the permeation, delivery and stabilization of ASO are major challenges in their gene therapeutic applications. Beyond integration of the ASO in polymeric nanoparticles, liposomes, lipid nanoparticles and DNA nanostructures, such as DNA origami or DNA tetrahedra, the chemical modification of the ASO with non‐nature nucleotides, resistant to the enzyme degradation, was employed to overcome the delivery and stability drawbacks. In the present section, the conjugation of ASO to nanoparticle carriers and the use of the hybrids as vehicles assisting cell permeation and therapeutic stability will be exemplified. Particularly, cooperative antisense/photothermal/photodynamic/chemotherapeutic activities of the conjugates for cancer therapy will be addressed.

**Figure 9:**
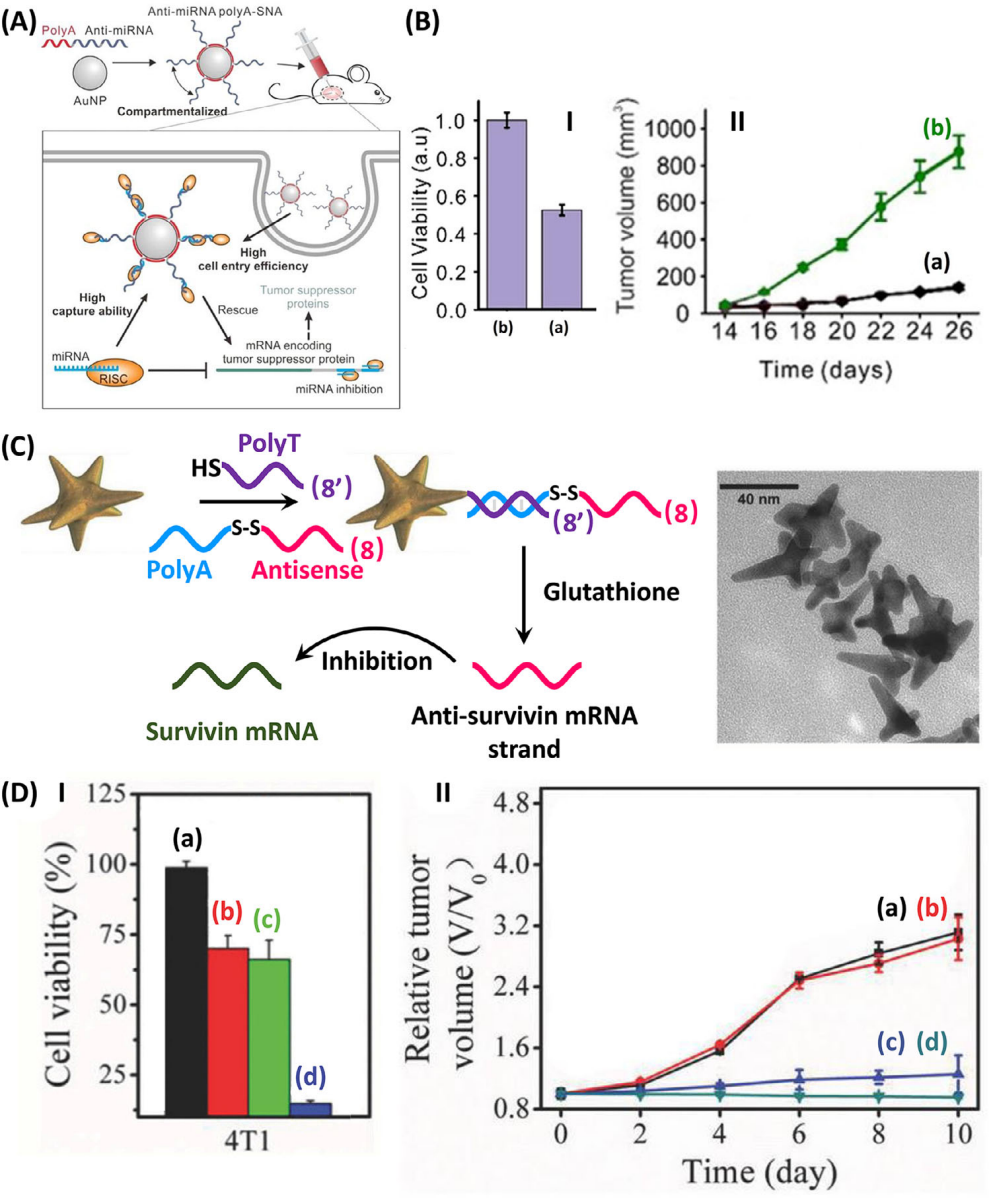
A) Schematic application of Au NPs functionalized with the anti‐miRNA strand that rescue the mRNA encoding tumor suppressor protein leading to cell apoptosis. B) Panel I‐MCF‐7 cell viability treated with the anti‐miRNA strand‐modified Au NPs (a) compared to non‐treated cells (b). Panel II‐MCF‐7 tumor growth inhibition upon treatment with the anti‐miRNA strand‐modified Au NPs (a) and non‐treated tumors (b). C) Schematic application of Au nanostars modified with disulfide‐caged anti‐survivin mRNA strands for the cooperative antisense silencing of anti‐survivin mRNA by GSH uncaging of the antisense sequence and photothermal treatment of the cells by plasmonic Au nanostars. D) Panel I‐Viability of 4T1 breast cancer cells: (a) non‐treated cells, (b) subjected to the anti‐survivin mRNA strand‐modified Au nanostars in the dark (only gene therapy), (c) subjected to random sequence‐modified Au nanostars under irradiation, λ_ex_ = 808 nm (only photothermal effect), (d) combined gene therapy and photothermal therapy by the anti‐survivin mRNA strand‐modified Au nanostars. Panel II‐Temporal 4T1 tumor growth inhibition upon treatment of the tumors at conditions ((a)–(d)) specified in Panel I. (A)‐(B) Reproduced with permission.^[^ ^163^ ^]^ Copyright 2021, Wiley. (C)–(D) Reproduced with permission.^[^ ^164^ ^]^ Copyright 2018, Wiley.

**Figure** **9**A exemplifies the antisense regulation of mRNA expression of the tumor suppressor protein thereby controlling apoptosis of cancer cells.^[^ ^163^ ^]^ The miRNA‐21 binding to Argonaute (Ago) forms the miRNA/RISC that binds to the mRNA encoding the tumor suppressor protein, resulting in cell apoptosis inhibition of cancer cells. Accordingly, Au nanoparticles were modified with polyA/anti‐miRNA‐21 conjugates as functional antisense agent rescuing the mRNA expression of the tumor suppressor protein. Permeation of the Au nanoparticle/antisense agent conjugate into MCF‐7 cancer cells blocks the miRNA‐21, thereby rescuing the activity of the tumor suppressor protein (Tropomyosin 1) resulting in the apoptotic activation of the tumor cells. Figure 9B, Panel I depicts the effect of the antisense agent‐modified Au nanoparticle on the MCF‐7 cell viability, ca. 53% cell death was demonstrated, while control conjugates including a scramble antisense sequence were inefficient. MCF‐7 breast tumor in xenograft mice treated with the antisense agent‐modified Au nanoparticles was inhibited by >95% the tumor growth as compared to control system, Panel II.

In a further example, Au nanostars modified with a disulfide linker bridged antisense strand (8), recognizing the survivin mRNA, were used for gene therapy of cancer cells, i.e., 4T1 breast cancer cells, Figure 9C.^[^ ^164^ ^]^ The (8)/(8’)‐modified Au nanostar hybrids underwent, in the presence of overexpressed glutathione in cancer cells, cleavage of the disulfide bridge releasing the antisense constituent. The intracellular release of the antisense agent inhibited the expression of survivin mRNA resulting in the enhanced apoptosis of the cancer cells. Moreover, the plasmonic photoexcitation of the Au nanostar assembly resulted in the cooperative photothermal heating of the cancer cells, leading to cooperative antisense‐induced and thermal destruction of the cancer cells. Figure 9D, Panel I demonstrated the antisense treatment of the 4T1 cells under dark conditions leading to ca. 30% cell death, and the glutathione‐stimulated release of a non‐antisense strand from the particle inducing under irradiation ca. 35% cell death (photothermally induced cell death). The cooperative photothermal effect and release of the antisense strand yield, however, a synergistic cell death corresponding to ca. 85%. The cooperative antisense/photothermal treatment of cancer cells with (8)/(8’)‐modified Au nanostar hybrids was further demonstrated with in vivo treating 4T1 tumor‐bearing mice, Figure 9D, Panel II. While effective tumor growth was observed in control mice samples treated with buffer solution or irradiated buffer solutions, significant inhibition of the tumor development treated with the (8)/(8’)‐modified Au nanostar particles in the dark or light was demonstrated.

**Figure 10:**
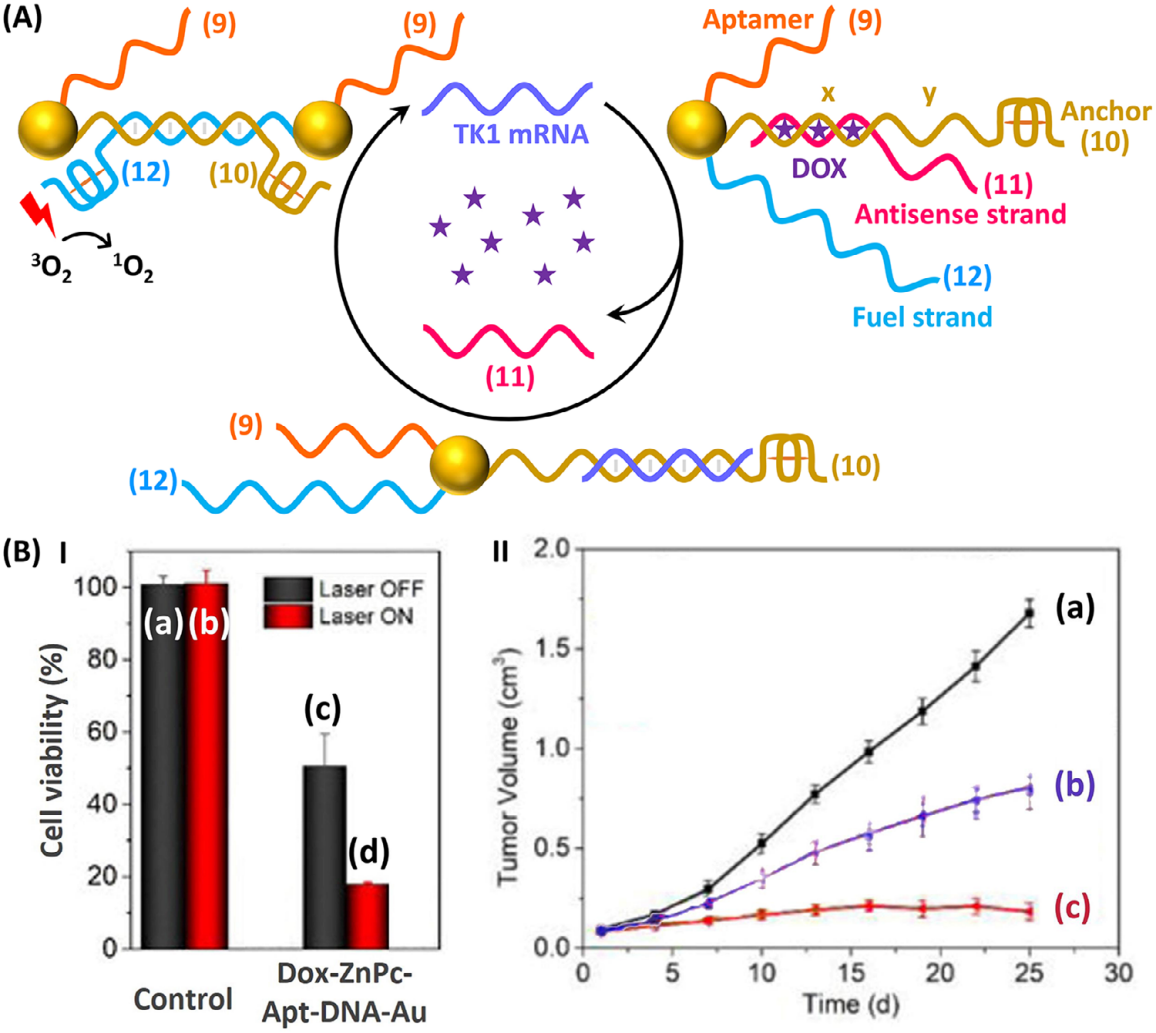
A) Schematic application of Au NPs functionalized with antisense/anchor duplex loaded with DOX and tethered to a G‐quadruplex, MUC1 aptamer and fuel strand for multimodal cooperative gene silencing, chemodynamic, photodynamic and photothermal treatment of cancer cells. B) Panel I‐MCF‐7 breast cancer cell viability: (a)/(b) in the absence of the functionalized Au NPs under dark/light conditions, (c) in the presence of the functionalized Au NPs in the dark, (d) in the presence of the functionalized Au NPs under light, λ_ex_ = 660 nm. Panel II‐Inhibition of MCF‐7 tumor growth: (a) in the absence of the functionalized Au NPs illuminated at 660 nm, (b) in the presence of the functionalized Au NPs without irradiation, (c) in the presence of the functionalized Au NPs under illumination, λ_ex_ = 660 nm. (A)‐(B) Reproduced with permission.^[^ ^165^ ^]^ Copyright 2021, Wiley.

Nucleic acid‐functionalized Au NPs for the targeted multimodal gene therapy, chemotherapy and photodynamic/photothermal therapy of MCF‐7 breast cancer cells are depicted in **Figure** **10**A.^[^ ^165^ ^]^ The Au NPs are functionalized with the anti‐MUC‐1 aptamer (9) as cell targeting constituent. The nucleic acid (10) acts as anchoring strand for hybridization of the anti‐survivin mRNA strand (11). The formation of the duplex between (10) and (11) provides a base‐pairing domain for intercalation of DOX chemotherapeutic agent. The anchoring strand (11) was pre‐engineered, however, to include the sequence “x”, complementary to part of the survivin mRNA, extended tether “y” complementary to part of the TK1 mRNA, and extended by a G‐quadruplex tether. In addition, a fuel strand (12) extended by a G‐quadruplex is co‐linked to the Au NPs. Upon anti‐MUC‐1 aptamer (9)‐targeted endocytosis of the hybrid, TK1 mRNA displaces the antisense strand (11) through hybridization to part of the domain “x”. Displacement of the antisense strand (11) results in its dissociation from the nanoparticle structure and release of the DOX drug. Moreover, the free strand (10) is pre‐engineered to hybridize with the fuel strand (12), resulting in the release of the TK1 mRNA and the aggregation of the Au NPs and the formed thermoplasmonic clusters, carrying ZnPc/G‐quadruplex unit as photodynamic ROS generating units. That is, the MUC‐1 aptamer‐assisted cellular uptake of the hybrid nucleic acid/Au NPs construct shown in Figure 10A leads to the autonomous release of the anti‐survivin mRNA oligonucleotide silencing survivin and the concomitant release of DOX as cooperative chemotherapeutic drug. The sequential events result in, however, the intracellular formation of aggregated Au NPs clusters, carrying ZnPc photosensitizer units, that upon photoirradiation lead to cooperative photodynamic and photothermal therapy of the cancer cells. While the cooperative gene therapy and chemodynamic therapy leads to ca. 50% cell death, the combined multimodal therapy involving gene therapy, chemotherapy, photodynamic and photothermal treatment results in 85% cell death, Figure 10B, Panel I. Similarly, in vivo experiment demonstrates the multimodal effects of the hybrid nanoparticles on the MCF‐7 tumor growth in mice, Figure 10B, Panel II. While the combined gene silencing/chemodynamic treatment inhibits tumor growth by 60%, the multimodal therapies consisting the gene therapy, chemotherapy, photodynamic and photothermal therapy, fully inhibit the tumor growth.

## 6 Conclusion

The review article addressed a rapidly developing topic in nanomedicine where oligonucleotide‐modified nanoparticle composites act as functional hybrids for cancer therapy. This topic was addressed and exemplified by discussing four different sub‐areas implementing the nucleic acid/nanoparticle composites for cancer therapy. **Table** **1** summarizes the sub‐topics addressed in the review article by the systematic identification of the nucleic acid functions and nature of the nanoparticle constituent in the therapeutic hybrids. Moreover, the cancer treatment (or cooperative treatment) methodologies driven by the hybrid structures and the detailed survey of types of cancer cells/tumors treated by the conjugates are listed. i) The use of aptamer‐functionalized nanoparticles for chemodynamic, photodynamic and photothermal treatment of cancer cells was discussed. The synergistic functions of the nanoparticle and aptamer constituents in the composite nanostructures were emphasized. These included the cooperative application of the catalytic properties of the nanoparticles yielding cytotoxic ROS products and the specific recognition properties of aptamer to target and facilitate selective permeation of the therapeutic catalysts into the cancer cells. Alternatively, the aptamer units linked to the nanoparticles acted as host units for supramolecular complex acting as photosensitizer generating cytotoxic ROS products for photodynamic treatment of the cancer cells. The nanoparticles, beyond acting as carriers for delivering the photoactive agents into the cancer cells, they often introduced cooperative photothermal therapeutic functionalities, e.g., through photochemical excitation of plasmonic carriers. ii) The use of catalytic and photocatalytic DNAzymes‐modified nanoparticles as composites for the generation of cytotoxic ROS species or catalyst for cleavage of mRNA associated with cancer was presented. Amplification means to enhance the intracellular photocatalysts and catalysts were described and methods to induce their selectivity toward cancer cells were addressed. iii) Methods to assemble stimuli‐responsive DNA‐gated anti‐cancer drug‐loaded nanoparticles were introduced. Particularly, the engineering of hybrid nanoparticles responding to biomarkers existing in cancer cells was addressed. These hybrid nanoparticles not only revealed autonomous drug‐release features in the presence of cancer cell biomarkers, but demonstrated selective activities in the cancer cells. iv) Within the broad efforts directed to the application of spherical nucleic acids (SNAs) as functional silencing or ASO carriers for cancer therapies, integration of nucleic acid‐modified nanoparticles for gene therapy is anticipated to have a significant impact. Indeed, siRNA and antisense oligonucleotide‐modified nanoparticles were introduced for gene therapy. The nanoparticles not only acted as carriers for the siRNA or antisense silencing agents, but synergistically participated in the cancer therapy by providing cooperative photodynamic and photothermal treatment pathways.

**Table 1:** Diverse application of nucleic acid/nanoparticle hybrids for cancer therapies.

Nucleic Acids	Nanoparticles	Cancer therapies	In vitro target	In vivo target	Reference
AS1411/MUC‐1 aptamer	Ce^4+^‐ion‐modified C‐dots	Chemodynamic	MDA‐MB‐231 breast cancer cells	MDA‐MB‐231 tumor in mice	[99]
AS1411 aptamer	Au nanoparticles	Chemodynamic	MDA‐MB‐231 breast cancer cells	MDA‐MB‐231 tumor in mice	[70]
Ce6‐modified PTK‐7 aptamer	Au nanorods	Photodynamic	Leukemia T‐cells	–	[100]
PTK‐7 aptamer	Zr^4+^‐Porphyrin framework	Photodynamic	Hela cancer cells	–	[101]
AS1411 aptamer	Au nanostars	photothermal	Hela cancer cells	–	[116]
AS1411 aptamer	Porphyrin/Graphene quantum dots (GQDs)	Photothermal Photodynamic	A549 cancer cells	–	[118]
MUC1 aptamer	CuS QDs and DOX‐loaded MOF‐199	Photothermal Chemotherapy	MCF‐7 cancer cells	MCF‐7 tumors in mice	[119]
Porphyrin/Q‐quadruplex (AS1411 aptamer)	Au nanoparticles	Photodynamic	HeLa cancer cells	HeLa tumors in mice	[135]
Porphyrin/G‐quadruplex and DOX‐intercalated duplexes	Au nanoparticles	Photothermal Photodynamic Chemotherapy	MDA‐MB‐231 breast cancer cells	MDA‐MB‐231 tumors in mice	[136]
G‐quadruplex (VEGF aptamer)‐DNA tetrahedra	Porphyrin‐loaded UiO‐66 MOF	Photodynamic	MDA‐MB‐231 breast cancer cells	‐	[137]
Ce6‐modified 10–23 DNAzyme	MnO_2_ particles	Photodynamic Gene therapy	MCF‐7 breast cancer cells	‐	[138]
Porphyrin/G‐quadruplex (AS1411 aptamer) and 10–23 DNAzyme	Upconversion nanoparticles	Photodynamic Gene therapy	MCF‐7 breast cancer cells	MCF‐7 tumors in mice	[139]
AS1411 aptamer and i‐motif‐modified DNA tetrahedra	DOX‐loaded UiO‐66 MOF	Chemotherapy	MDA‐MB‐231 breast cancer cells	‐	[140]
mi‐RNAs responsive DNA duplexes	UiO‐68 MOF	Chemotherapy	OVCAR‐3 cancer cells, MCF‐7 breast cancer cells	‐	[141]
G‐quadruplex (VEGF aptamer)‐DNA tetrahedra	UiO‐66 MOF	Chemotherapy	MDA‐MB‐231 breast cancer cells	‐	[137]
Bcl‐2 siRNAs	COOH/NH_2_ and guanidinium‐functionalized C‐dots	Gene therapy	HeLa or MCF‐7 breast cancer cells	HeLa tumors in mice	[160]
NGF siRNAs	Au nanoclusters	Gene therapy	Panc‐1 cancer cells	Panc‐1 tumors in mice	[161]
Bcl‐2 siRNAs	Cu^2+^‐ion‐modified layered double hydroxide nanoparticles	Gene therapy Photothermal	B16F0 melanoma cancer cells	B16F0 tumors in mice	[162]
anti‐miRNA‐21 ASOs	Au nanoparticles	Gene therapy	MCF‐7 cancer cells	MCF‐7 breast tumors in mice	[163]
Anti‐survivin mRNA ASOs	Au nanostars	Gene therapy Photothermal	4T1 cancer cells	4T1 tumors in mice	[164]
MUC1 aptamer, anti‐survivin mRNA ASOs, DOX‐intercalated DNA duplexes, porphyrin/G‐quadruplex	Au nanoparticles	Gene therapy Chemotherapy Photodynamic Photothermal	MCF‐7 breast cancer cells	MCF‐7 breast tumors in mice	[165]

Throughout the review article, we discussed fundamental concepts applying the nucleic acid‐modified nanoparticles for cancer therapy. We emphasized the use of diverse nanoparticle materials, the versatility of oligonucleotide functions in the hybrid structures, guided by the structural and functional information encoded in the oligonucleotides. Moreover, the significance of the cooperative, multimodal functionalities of the hybrid nanostructures was emphasized.

The review article summarized the advances in developing functional nucleic acid‐modified nanoparticles for cancer therapy. While important scientific accomplishments were preceded, many future challenges need to be addressed to further develop these composite materials and translate the hybrid framework for clinical use. For example, the study emphasized the implementation of doxorubicin‐loaded carriers for chemotherapeutic treatment of cancer cells. For many other chemotherapeutic drugs,^[^ ^166^ ^]^ such as cisplatin, 5‐fluorouracil, barriers in the loading of the different drugs in the carriers could be faced, and a fair therapeutic efficacy of the different drugs (or composition of drugs) is essential for future applications. Moreover, throughout the report, the selectivity of the carriers toward the cancer cells vs. normal epithelial cells has been emphasized. Nevertheless, the selectivity could originate from either different permeabilities of the carriers to the different type of cells or different intracellular biomarkers, activating the release of the loads from the carriers, in the different types of cells. These parameters are often overlooked upon analyzing the selectivity issues. It is important to discriminate these parameters in any future studies. Furthermore, the effective intracellular escape of the functional hybrids from the delivered endosomal containments into the cytosol environment is a major parameter regulating their therapeutic efficacy.^[^ ^167^, ^168^ ^]^ While different modifying ligands of the hybrids, such as amino acids,^[^ ^160^ ^]^ peptides,^[^ ^169^ ^]^ or polymer sponges,^[^ ^170^ ^]^ were reported to assist the endosomal escape process, development of additional escape promotors and elucidation of the escape mechanisms are important.

The translation of the fundamental scientific advances in developing nucleic acid/nanoparticle hybrids for clinical applications presents important scientific and technological future goal, yet facing significant future challenges. At present, spherical nucleic acids (SNAs) structures were reported as effective diagnostic nanotools and approved for clinical gene analysis. Moreover, diverse SNAs‐based therapeutics are at different phases of clinical evaluation for diverse diseases including cancer. These advances were recently addressed in several review articles.^[^ ^89^, ^90^, ^171^ ^]^ The translation of nucleic acid‐modified nanoparticles for clinical evaluation is certainly close to realization. Nevertheless, the roadblocks of clinical translation should take into account, as pointed out recently,^[^ ^172^, ^173^ ^]^ immunotoxic effects associated with these innovative therapeutic platforms.

The progress applying oligonucleotide/nanoparticle hybrids for nanomedicine, particularly cancer therapy, were demonstrated, yet substantial future challenging developments may be envisaged. Continuous efforts in nanotechnology promotes the development of new functional nanomaterials. For example, nanomaterials revealing superior catalytic, photocatalytic and photothermal effects were reported, and nanoparticles exhibiting plasmonic “hot” spots, such as Au triangle or pyramid structures^[^ ^174^ ^]^ were reported. Sonodynamic, ultrasound‐stimulated activation of catalytic functions of nanoparticles,^[^ ^175^ ^]^ magnetic field‐induced catalytic activities of magnetic materials,^[^ ^176^ ^]^ and nanoparticle‐mediated radiotherapy enhancing antitumor efficacy^[^ ^177^ ^]^ were reported. Also, inorganic composite nanomaterials, such as MXene,^[^ ^178^ ^]^ demonstrated superior photothermal properties. The conjugation of functional nucleic acids to these nanoparticle‐based therapies could overcome cell permeation barriers and limited selectivity accompanying those therapeutic platforms. Moreover, the limited permeation of nanoparticles into cells/tissues and their targeting to the diseased cells for controlled selective, spatiotemporal treatment is a key issue for their medical applications. The unique cell permeation efficacies of DNA tetrahedra nanostructures,^[^ ^179^ ^]^ their tunable 3D,^[^ ^180^ ^]^ their further modification with DNAzymes or aptamer tethers,^[^ ^181^ ^]^ and the synergistic feasibility to modify nanoparticles with DNA tetrahedra frameworks^[^ ^154^ ^]^ provide versatile means to apply oligonucleotide/nanoparticle hybrids for therapeutic uses. Finally, the information encoded in nucleic acid nanostructures has been broadly implemented to diverse gene therapies, such as the engineering of DNA‐based carriers for immunogenic therapy or the CRISPR/Cas gene editing therapy. Coupling these advanced gene therapeutic frameworks to functional catalytic nanoparticle systems is anticipated to introduce innovative new dimensions for nanomedicine, particularly cancer therapy.

## Conflict of Interest

The authors declare no conflict of interest.
